# Gut microbes and immunotherapy for non-small cell lung cancer: a systematic review

**DOI:** 10.3389/fonc.2025.1518474

**Published:** 2025-05-08

**Authors:** Yali Jin, Zhiqian Jie, Xianming Fan

**Affiliations:** ^1^ Department of Respiratory and Critical Care Medicine, The Affiliated Hospital of Southwest Medical University, Luzhou, Sichuan, China; ^2^ Inflammation and Allergic Diseases Research Unit, The Affiliated Hospital of Southwest Medical University, Luzhou, Sichuan, China

**Keywords:** immunotherapy, gut microbiota, non-small cell lung cancer, immune checkpoint inhibitors, dietary intervention

## Abstract

Emerging evidence underscores gut microbiota’s role in modulating lung cancer immunotherapy outcomes, though specific impacts on immune checkpoint inhibitors (ICIs) and associated adverse events (AEs) require further clarity. This review synthesizes findings from 15 studies examining gut microbiota-ICI interactions in non-small cell lung cancer (NSCLC), alongside studies investigating antibiotics, proton pump inhibitors (PPIs), probiotics, and diet as modulating factors. Results indicate that *Actinobacteria*, *Bacteroides*, and *Verrucomicrobiota* correlate with positive ICI responses, while *Bacillota* shows variable associations; notably, *Bacillota*-enriched patients had fewer immunotherapy-related AEs. The administration of antibiotics and PPIs within a month before ICIs was linked to diminished efficacy, whereas probiotics correlated with enhanced outcomes. Plant-based diets are also aligned with dietary patterns supportive of ICIs. These findings suggest that analyzing gut microbiota composition could improve the ability to predict NSCLC patient responses to ICIs. Additionally, judicious use of antibiotics, PPIs, probiotics, and dietary adjustments may optimize immunotherapy outcomes and mitigate adverse effects.

## 1 Introduction

Lung cancer is the most prevalent cancer worldwide, accounting for 11.6% of all cases. Even more concerning, it is responsible for 18.4% of cancer-related deaths. Among the different types, non-small cell lung cancer (NSCLC) is the most common (1). The emergence of immune checkpoint inhibitors (ICIs) has provided a new treatment approach for NSCLC patients (2). However, this treatment is not without adverse effects (AEs), which can impede the therapeutic efficacy in some patients (3). Recent research has shown that gut microbiota significantly influences the effectiveness of immunotherapy in NSCLC (4). The development and function of immune cells are influenced by gut microbiota on immune responses (5). The effectiveness and side effects of ICIs are closely associated with the composition and diversity of gut microbiota (6). However, there has been little research on ICIs’ effect on gut microbiota in NSCLC patients. We intend to synthesize research regarding the influence of the gut microbiota on treating non-small cell lung cancer (NSCLC) with immune checkpoint inhibitors (ICIs), including potential factors that may impact outcomes. The findings will provide valuable references for future clinical practice and therapeutic strategies.

## 2 Materials and methods

This study follows the PRISMA guidelines (7).Inclusion and exclusion criteria: The study population included NSCLC patients; observational studies providing information on the efficacy and/or adverse effects of gut flora, antibiotics, PPIs, probiotics, and diet in relation to immunotherapy for non-small cell lung cancer were included, while case reports, reviews, conference proceedings, and abstracts were excluded. Search Strategy:Searches of PubMed, Embase, and Web of Science databases were conducted to include English-language studies from the time of construction through March 26, 2024. Searches were conducted using the subject terms “carcinoma, non-small cell lung,” “gut microbiome,” “microbiota,” “antibiotics,” “proton pump inhibitors,” “diet,” “healthy adult,” “probiotics,” “immunotherapy,” and related free keywords. The initial screening of studies was performed by two authors on the basis of the title and abstract, followed by a careful reading of the full article, and in case of disagreement, the decision was discussed with a third author (**Supplementary Files**). Quality assessment: Risk of bias was assessed using the Newcastle-Ottawa Scale (NOS) with a score ranging from 0-9 (8). Studies were subsequently categorized as low-moderate quality (score <7) and high quality (score ≥7) based on their quality scores. Data extraction: two authors extracted data using a predefined form and discussed with the third author in case of disagreement. Data extraction included: 1st author, year of publication, study design, country of study, sample size, patient characteristics, gut flora sequencing methods, and flora characteristics. Statistical analysis: descriptive statistics were used to summarize the results of the study and Fisher’s test was used to assess the differences between the two groups, with P < 0.05 indicating statistical significance.

## 3 Results

### 3.1 Gut microbiota is associated with response to ICIs and adverse events in NSCLC

**Figure 1:**
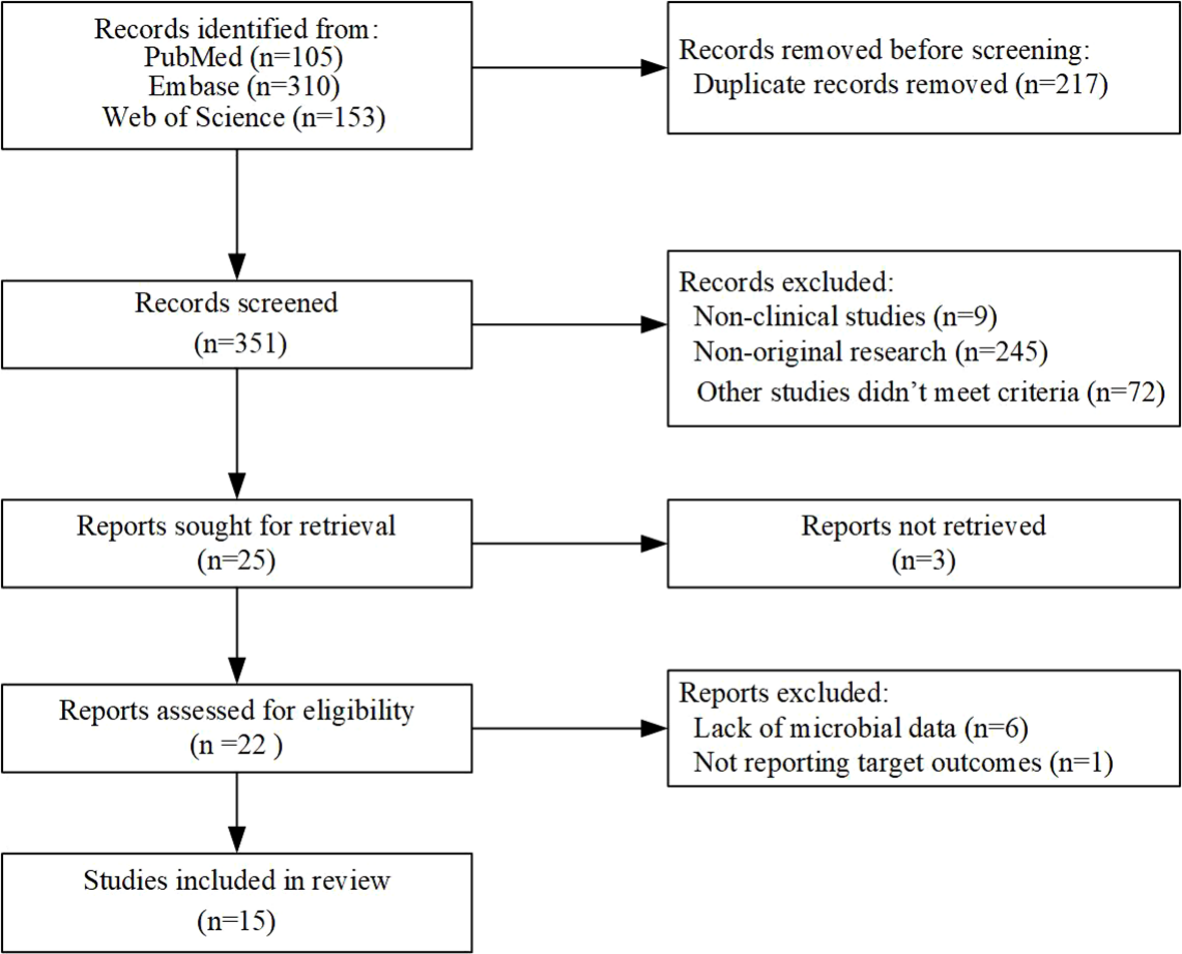
Flowchart of eligible studies based on the ICI for gut microbiota and non-small cell lung cancer.

This study focused on exploring the connection between intestinal microflora and the therapeutic outcomes of immune checkpoint inhibitors (ICIs) in non-small cell lung cancer (NSCLC). A comprehensive search was conducted using the keywords “carcinoma, non-small-cell lung,” “gut microbiome,” and “immunotherapy,” which yielded 568 potentially eligible studies. After rigorous manual screening, 15 clinical studies related to the gut microbiota and immune checkpoint inhibitor (ICI) therapy in non-small cell lung cancer (NSCLC) were identified, with the majority of these studies being of high quality (**Figure 1**). With 15 of the studies having data on treatment response to ICIs and 5 of them having data on both treatment response to ICIs and immune-related adverse effects (AEs) (4, 6, 9–21). The research on the association between gut microbiota and response to immunotherapy in NSCLC included a total of 763 patients, primarily from China, Spain, Poland, Hungary, and Japan, with ages ranging from 30 to 88 years. Additionally, 358 patients, mainly from Spain, Hungary, and Japan, aged between 31 and 88 years, were included to assess the correlation between gut microbiota and immune-related AEs associated with NSCLC immunotherapy (**Supplementary Tables**).

**Figure 2:**
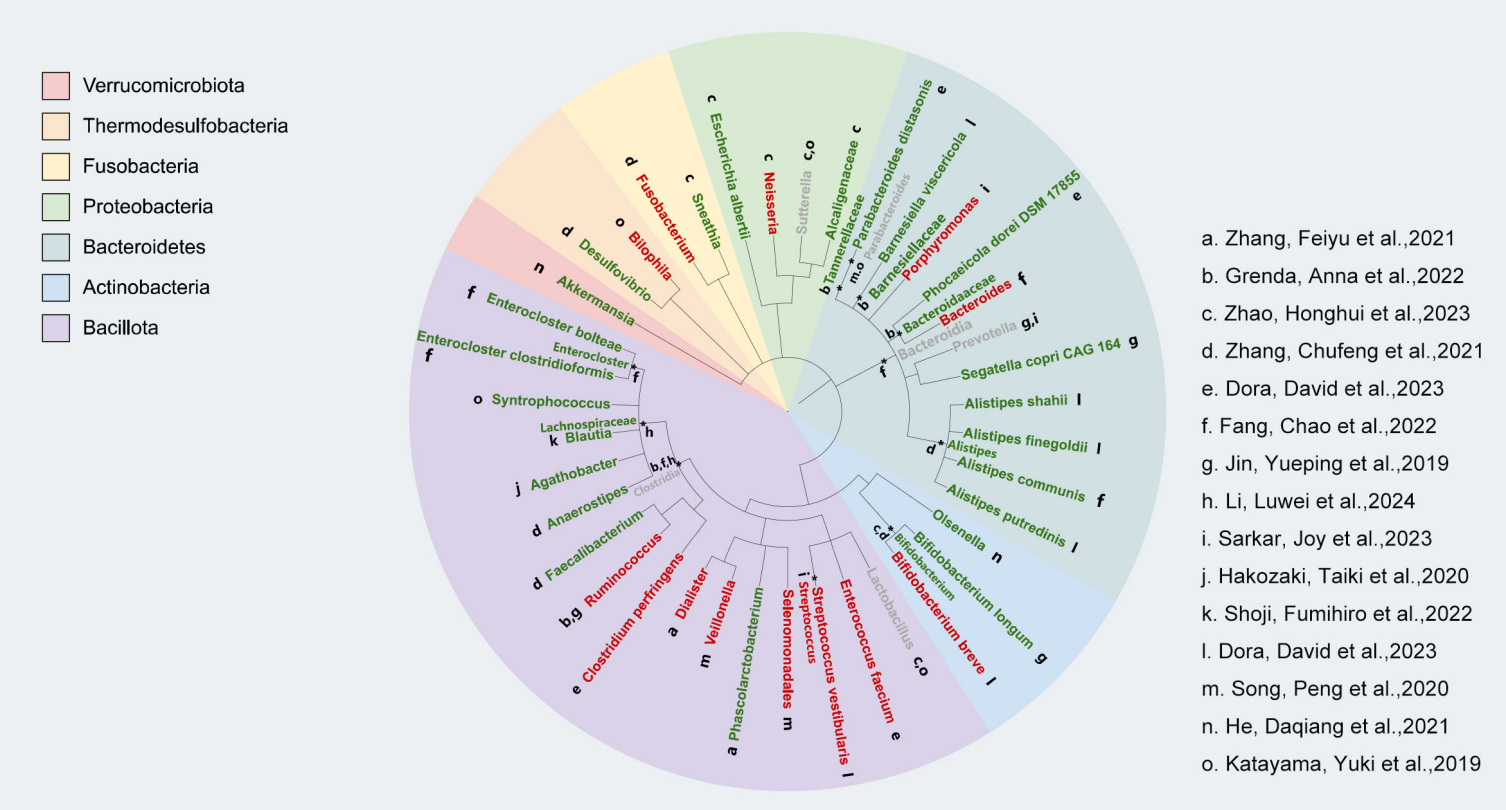
Relationship between gut microbiota and the response of NSCLC patients to treatment with immune checkpoint inhibitors (ICI). Individual studies generated from bacterial taxon information are represented by lowercase letters next to each bacterium; better response is indicated by green markers, poor response is indicated by red markers, and mixed response is indicated by gray markers.

**Figure 3:**
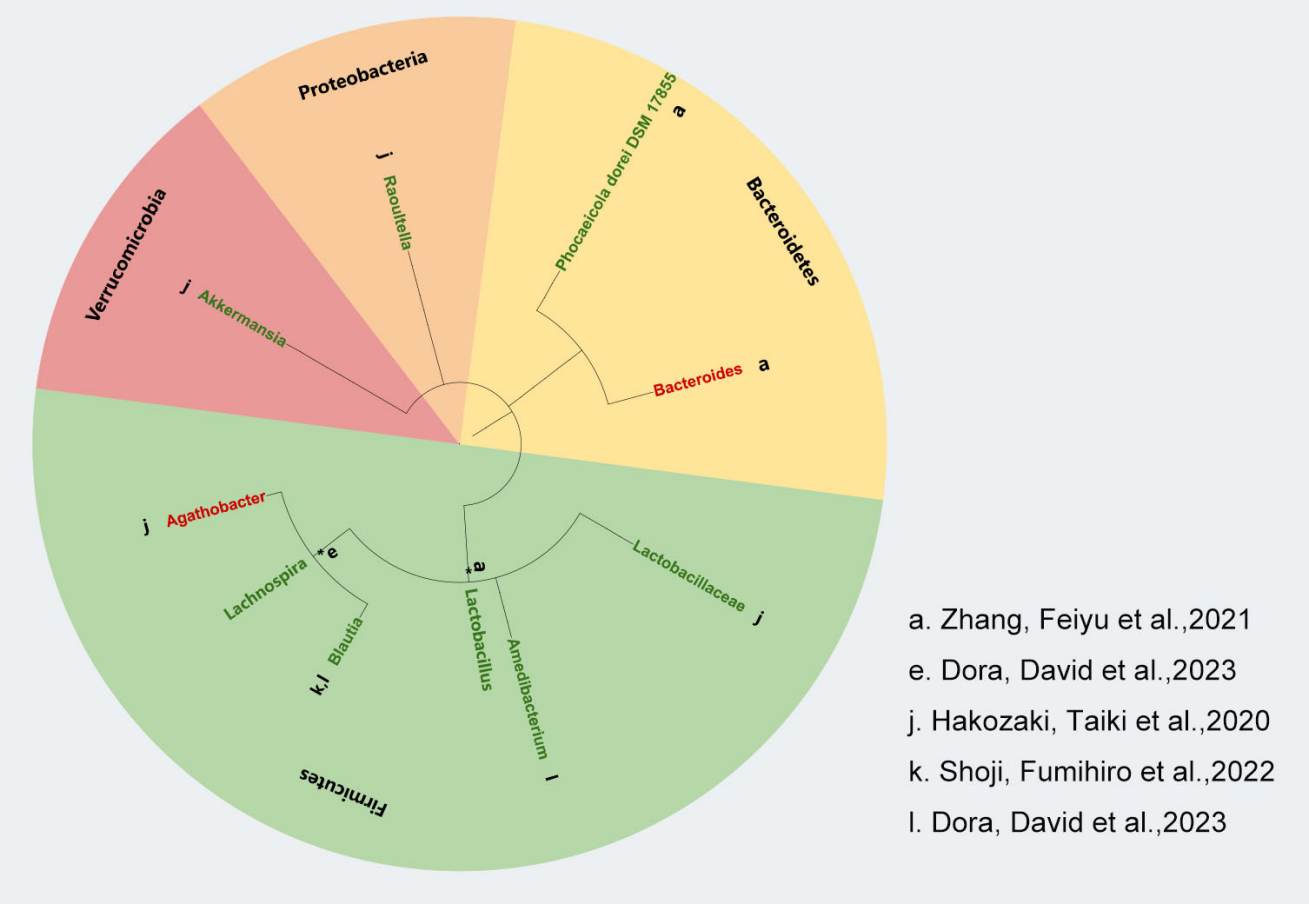
Correlation between gut microbiota and AEs to ICIs in NSCLC. Lowercase letters next to each bacterial species represent distinct studies based on bacterial taxon data. Green markers indicate fewer adverse reactions, red markers indicate more adverse reactions and black asterisks (*) indicate bacterial taxa identified at the genus level.

We used the phyloT online tool (https://phylot.biobyte.de) to map and evaluate the gut microbiota data that we gathered from pertinent studies. As shown in **Figure 2**, our analysis revealed that a higher abundance of *Actinobacteria*, *Bacteroidetes*, and *Verrucomicrobiota* was linked to positive clinical outcomes in ICI treatment. However, the response to *Bacillota* varied, with some inconsistent findings across two studies. Additionally, we noted that *Proteobacteria* might be related to a favorable response to immunotherapy efficacy. Not only that but in our AEs study on the gut microbiota and ICIs, we found that almost all enriched *Bacillota* were associated with a reduced incidence of AEs to immunotherapy, whereas no significant differential changes were observed in the *Bacteroidetes* (**Figure 3**).

### 3.2 Impact of antibiotics on treatment of ICIs in NSCLC

**Figure 4:**
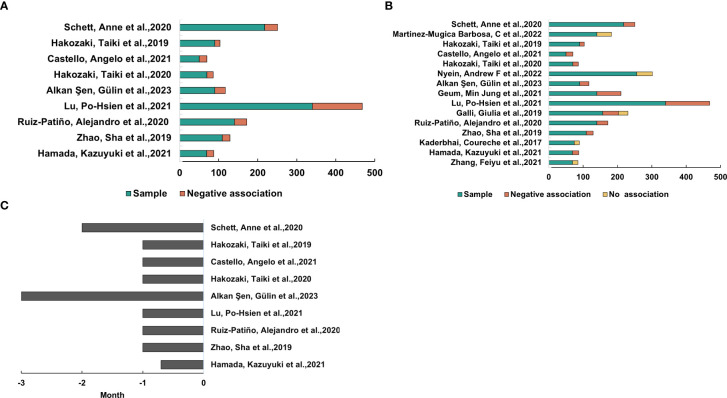
Antibiotic exposure’s impact on ICIs’ effectiveness for NSCLC. **(A)** Research shows that using antibiotics within a month of starting ICI treatment was linked to worse clinical results. **(B)** Studies in which the outcome of ICI therapy for NSCLC was adversely affected or did not correlate with antibiotic use. **(C)** The amount of time that the trials in **(B)** used antibiotics.

From the analysis above, we discovered an association of gut microbiota with the treatment efficacy of ICIs in NSCLC. This finding prompted the hypothesis that antibiotics, as modulators of gut flora, could influence the effectiveness of ICIs in NSCLC. To investigate the impact of antibiotics on ICI efficacy, a search was conducted using the keywords “antibiotics,” “immunotherapy,” “microbiota,” and “carcinoma, non-small-cell lung.” Following screening, 15 relevant studies were identified (**Supplementary Figure S1**). Out of 2012 patients assessed, 570 were undergoing antibiotic treatment. Two of the studies were prospective (6, 17), while the remaining 13 studies were retrospective (22–34). As illustrated in **Figure 4A**, most studies indicated that antibiotic use negatively impacted ICI treatment efficacy, although five studies reported no significant effect. We noted differences in outcomes across various studies based on antibiotic use before or after ICI treatment, suggesting that the timing of antibiotic administration might affect the efficacy of ICI therapy (**Supplementary Figure S2**). To explore this further, we further analyzed the timing of antibiotic use and the effect on outcomes, extracted the studies with negative effects, and got the conclusion that antibiotic use 1 month before ICIs treatment had a negative effect (**Figures 4B, C**).

### 3.3 Influence of proton pump inhibitors and probiotics on the effectiveness of immune checkpoint inhibitors in NSCLC

Proton pump inhibitors may disturb the ecological equilibrium of gut microbiota through the suppression of gastric acid secretion, potentially affecting the effectiveness of ICIs. To assess the impact of PPIs on ICI effectiveness in treating NSCLC, a literature search was conducted using key terms such as “proton pump inhibitors,” “immunotherapy,” “microbiota,” and “carcinoma, non-small-cell lung.” A total of five relevant studies were identified (35–39), all of which were retrospective studies, two of which were from references (36, 37) (**Supplementary Figure S3**). Three studies indicated that PPI use may negatively affect ICI treatment outcomes, with the adverse effects primarily occurring within one month prior to ICI therapy. Notably, one study did not specify the timing of PPI use, and thus, descriptive analysis of its timing was not conducted (**Supplementary Figure S4**).

Probiotics, which are live bacteria that are beneficial to the body, improve the activity of intestinal microorganisms and may influence the efficacy of ICIs. To determine if probiotics could improve the effectiveness of ICIs in treating NSCLC, a search was conducted using the keywords “probiotics,” “immunotherapy,” and “carcinoma, non-small-cell lung.” This search identified six relevant clinical studies (40–45), two of which were from references (41, 44) (**Supplementary Figure S5**). The study populations, comprising a total of 1,711 participants, were based in Japan, the United States, and Europe, with 12.5% of participants using probiotics. Four studies indicated that probiotics enhanced ICI efficacy, significantly improving both progression-free survival (PFS) and overall survival (OS) in patients (**Supplementary Figure S6**).

### 3.4 Potential impact of diet on the treatment of ICIs in NSCLC

**Figure 5:**
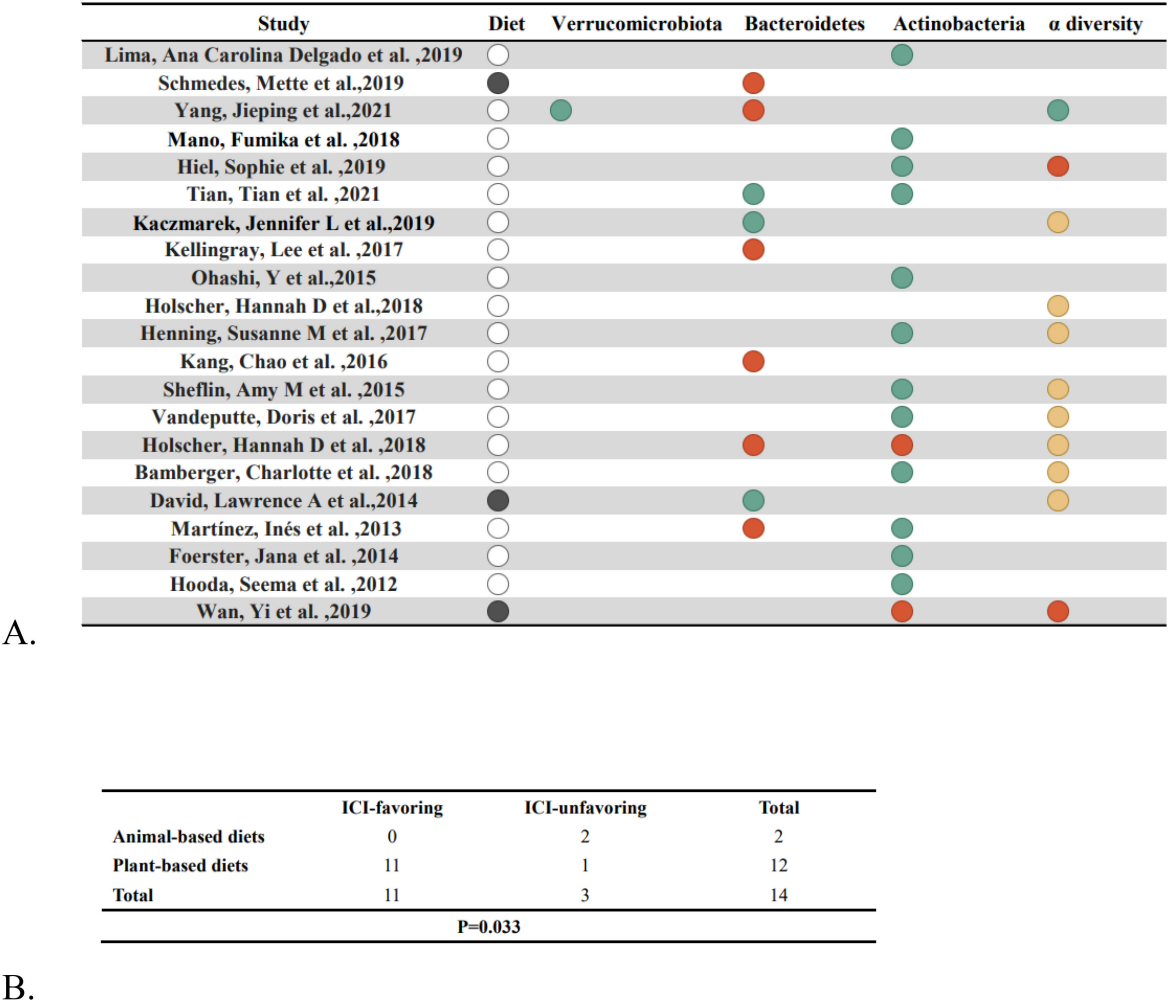
Nutritional factors and gut flora. **(A)** After dietary intervention, changes in gut bacteria are shown. Solid black circles indicate an animal diet, blank circles indicate a plant diet, green circles indicate an increase, red circles indicate a drop and yellow circles indicate no change. **(B)** Fisher’s exact test (p=.033) compares the enrichment of “ICI-favorable” and “ICI-unfavorable” gut microorganisms in response to plant-based vs. animal-based diets.

We initially searched the literature using the keywords “diet,” “gut microbiome,” “immunotherapy,” and “carcinoma, non-small-cell lung.” However, it was difficult to obtain a sufficient number of studies, so we sought to investigate the link between diet and the gut microbiome by employing the keywords “diet”, “gut microbiome”, and “healthy adult”. We retrieved 671 studies, and 17 results were identified after strict qualification screening (46–62), and another 4 results were from studies in the references (63–66), for a total of 21 studies (**Supplementary Figure S7**). According to our earlier findings, we identified *Actinobacteria*, *Bacteroidetes*, and *Verrucomicrobiota* as well as a rise in α-diversity as diets favorable to the treatment of those ICIs, while diets unfavorable to the treatment of those ICIs produced the reverse effect. The 21 studies covered a total of 820 study participants from the countries of the United States, Brazil, Norway, China, Japan, Belgium, the United Kingdom, and Germany, with a predominance of females, aged 18–72.4 years, and a BMI of 18.6-36.6 kg/m2. We divide our food intake into two categories: plant-based (vegetables, fruits, whole grains, nuts, etc.) and animal-based (meat, eggs, milk, etc.). The results are shown in **Figure 5A**, where we identified 11 out of 18 studies that were supportive of plant-based diets and one study that was not supportive, compared to two studies based on three animal-based diets, leading to the conclusion that plant-based diets were significantly related to favoring the treatment of ICIs (**Figure 5B**) (p = .033).

## 4 Discussion

While the advent of immune checkpoint inhibitors (ICIs) has greatly changed the treatment for NSCLC, challenges such as drug resistance and immunotherapy-related side effects have limited their potential to achieve optimal efficacy (67). Gut microbiota is vital in regulating the immune response during cancer progression. Intratumoral microbiota interact with pattern recognition receptors (PRRs) to evade immune surveillance; however, they also stimulate immune cell production and boost immune function, ultimately enhancing the effectiveness of immunotherapy (68). Some studies have shown that fecal bacterial transplantation can improve resistance to tumor immunotherapy, which is associated with changes in the intestinal microbiota and tumor microenvironment (69). This research investigates the link between gut microbiota and the management of non-small cell lung cancer using ICIs, focusing on the impact of gut modulation and diet on the effectiveness of ICIs. The findings aim to provide valuable insights for improving clinical treatment outcomes.

The clinical efficacy of immune checkpoint inhibitors (ICIs) in patients with non-small cell lung cancer (NSCLC) is influenced by the composition of the gut microbiota. We found that higher abundances of *Actinobacteria*, *Bacteroidetes*, and *Verrucomicrobia* were associated with better efficacy of ICIs, while the role of Firmicutes in ICI efficacy is more complex, showing both promoting and inhibiting effects. *Bifidobacterium*, a genus within the phylum *Actinobacteria*, has been shown to enhance the efficacy of ICI treatment in NSCLC patients.A study suggests that *Bifidobacterium* enhances the anti-tumor effect of anti-PD-L1 antibodies by promoting dendritic cell function and increasing the accumulation of CD8+ T cells in the tumor microenvironment (70). Another preclinical study showed that *Bifidobacterium* can reduce tumor burden in mice by enhancing immune responses, possibly through the biosynthesis of immune-stimulatory molecules and metabolic products (71). However, other studies have found that an increase in the abundance of *Actinobacteria* is associated with shorter progression-free survival (PFS) in NSCLC patients receiving ICI treatment, suggesting that the specific mechanisms of *Actinobacteria* in ICI therapy still require further investigation (20). Gopalakrishnan et al. found that *Bacteroidetes* is closely associated with a favorable response to PD-1 inhibitors, as *Bacteroidetes* modulates the immune microenvironment to activate anti-tumor T cell responses (72). In our study, we also found an enrichment of *Alistipes putredinis* and *Parabacteroides distasonis* in NSCLC patients who had a good response to ICIs. Rebecca L. Brown et al. proposed that *Bacteroidetes* exhibit strong immune-stimulating abilities through the activation of TLR4 (Toll-like receptor 4), and their effects on gut immunity are almost entirely dependent on the TLR4 receptor (73). In addition, *Verrucomicrobia*, such as *Akkermansia muciniphila*, has been shown to enhance the efficacy of immune therapy, particularly by improving gut barrier function and increasing immune cell infiltration, thereby boosting the patient’s response to immunotherapy (74). Studies have shown that oral administration of *Akkermansia muciniphila* can increase the recruitment of CCR9+CXCR3+CD4+ T lymphocytes to the tumor bed, thereby restoring the efficacy of PD-1 inhibitors in NSCLC patients (74). The role of *Firmicutes* in ICI treatment is more complex and may be influenced by factors such as sample size, patient population characteristics (e.g., age, sex, disease type), and variations in sequencing methods. An observational study found that *Clostridium butyricum* MIYAIRI 588 strain (CBM588) significantly prolonged the overall survival of NSCLC patients receiving combined chemotherapy and immunotherapy. This effect may be related to CBM588’s ability to expand the population of resident *Bifidobacterium*, thereby promoting anti-tumor immunity and enhancing ICI efficacy (75, 76). On the other hand, a study involving 67 advanced NSCLC patients found that an increase in the *Ruminococcaceae* family was associated with a lack of response to immunotherapy (77). Additionally, *Firmicutes* has been linked to a reduction in immune therapy-related adverse effects. Certain strains, such as *Faecalibacterium prausnitzii*, possess immune-regulatory and anti-inflammatory properties, likely influencing the host’s immune response through the production of short-chain fatty acids, such as butyrate. This may help alleviate immune therapy-related side effects, such as colitis induced by immune checkpoint inhibitors (72). However, a study by Dubin et al. showed that an increase in certain bacterial populations within the *Bacteroidetes* phylum is associated with resistance to checkpoint blockade-induced colitis, which complicates the understanding of the gut microbiota’s role in modulating the effects of immunotherapy (78). In addition, some studies have suggested that higher alpha diversity may be associated with better immunotherapeutic response, suggesting that gut microbial diversity may influence immune system function and thus interact with the efficacy of immune checkpoint inhibition therapies (4). However, this finding has not been consistently supported across studies, and some studies have failed to find a clear association between alpha diversity and immune efficacy. In conclusion, the role of the gut microbiota in the efficacy of ICIs in NSCLC patients is multifaceted, and further research is needed to uncover its underlying mechanisms.

Antibiotic use reduces the efficacy of immune checkpoint inhibitors (ICIs), particularly negatively affecting overall survival (OS) and progression-free survival (PFS) in patients with non-small cell lung cancer (NSCLC). The study demonstrated that patients with non-small cell lung cancer (NSCLC) who received antibiotic treatment within one month prior to the commencement of immunotherapy exhibited significantly poorer outcomes. This finding aligns with the observations reported in a study by DeRosa et al., which noted a substantial decline in overall survival (OS) among NSCLC patients who received β-lactam or quinolone antibiotics, particularly within the initial 30 days of treatment (79). However, research on the long-term effects of antibiotics on gut microbiota and ICI outcomes remains limited. The question of whether antibiotics exert temporary or permanent effects on the gut microbiota is still under debate. A study by Huang et al. used 16S rRNA gene sequencing to assess the short- and long-term effects of ampicillin, vancomycin, metronidazole, and neomycin on the gut microbiota in mice. The results revealed that these oral antibiotics have a long-lasting negative impact on the gut microbiota, promoting the proliferation of antibiotic-resistant strains and causing irreversible changes in microbial diversity (80). Nevertheless, the specific impact of these changes on the long-term efficacy of immunotherapy remains unclear, and longer follow-up studies are needed in the future to explore this further. Furthermore, an investigation was conducted into the impact of proton pump inhibitors (PPIs) on patients with non-small cell lung cancer (NSCLC) undergoing treatment with immune checkpoint inhibitors (ICIs), with particular emphasis on the potential adverse effects of PPI utilization. This effect may be related to the alteration of the gut microbiota due to PPIs, thus affecting the pharmacokinetics and pharmacodynamics of ICIs. However, previous studies have shown no significant association between PPI use and overall survival (OS) or progression-free survival (PFS) in NSCLC patients treated with ICIs (81). These discrepancies may be attributable to a range of factors, including ambiguous time frames for PPI exposure or the presence of other potential confounders that have not been sufficiently addressed. Consequently, there is a necessity for more sophisticated study designs and a more comprehensive consideration of influential factors when analyzing the effects of PPIs on the efficacy of ICIs. The paucity of research on the long-term effects of PPIs on the gut microbiota and the efficacy of ICIs is a key area for future investigation. The long-term use of PPIs may continue to alter the pH of the gastrointestinal tract, which in turn affects the ecological balance of the intestinal microbiota. However, further investigation is required to fully elucidate the long-term effects of PPIs on the intestinal microbiota and the efficacy of ICIs. The favorable link between probiotic use and immunotherapy outcomes can be attributed to their ability to beneficially modulate the gut microbiota. Studies suggesting that specific gut microbiota (e.g., Bifidobacterium) may enhance antitumor responses to anti-PD-1 therapy have been reported by Sivan et al. and Vetizou et al (82, 83). Additionally, a study by Matson et al. noted that specific gut microbiota are significantly associated with immunotherapy efficacy, and these microbiota can often be enhanced by probiotic supplementation (84).

There is a growing recognition of the complex relationship between gut microbiota and nutrition in regulating the immune system and tumor microenvironment. Our findings identify potential benefits of plant-based diets in enhancing the efficacy of immune checkpoint inhibitors (ICIs). Specifically, plant-based diets can increase the abundance of *Actinobacteria*, *Bacteroidetes*, and *Verrucomicrobia*, while also enhancing the alpha diversity of the gut microbiota, thereby improving the efficacy of ICIs. On the other hand, animal-based diets can reduce the abundance of these beneficial bacteria, negatively affecting the efficacy of ICIs. Recent studies support our findings and suggest that a high-fiber, plant-based diet may enhance the therapeutic effects of ICIs by promoting the proliferation of beneficial gut microbiota. For example, one study recommended that patients consume at least thirty different plant-based foods each week, such as nuts, seeds, grains, fruits and vegetables, to optimize the effectiveness of ICIs (85). This dietary approach may indirectly affect the tumor microenvironment by enhancing intestinal barrier function and modulating the immune system, while providing essential nutritional substrates for the gut microbiota. However, it is important to note that the conclusion of this review that a plant-based diet improves the efficacy of ICIs is largely based on observational studies, which can themselves be affected by potential confounding factors. First, people who choose to follow a plant-based diet tend to be in better overall health and may be more attentive to lifestyle factors such as exercise and sleep, all of which can affect the efficacy of immunotherapy. For example, Gustafson et al. showed that exercise and physical activity can improve immunotherapy outcomes in cancer patients (86). Thus, lifestyle factors may to some extent influence the observed association between a plant-based diet and the efficacy of ICIs. Second, some individuals who choose a plant-based diet may also supplement with probiotics, which may themselves have a beneficial effect on immunotherapy by modulating the gut microbiota and enhancing immune responses. Thus, the use of probiotics may also be an important confounding factor in the efficacy of immunotherapy. Although certain dietary patterns, such as the Mediterranean diet or the ketogenic diet, have been shown to improve the response to immunotherapy, personalized dietary recommendations may be more helpful in improving the response to ICI due to individual differences in diet-induced changes in the microbiota (87, 88).Individuals respond differently to the gut microbiota of the same diet, which may be related to the individual’s gut environment and lifestyle factors. At present, research into the effect of personalized nutrition on immunotherapy is still in its early stages and the evidence is limited. Future studies can improve the effectiveness of immunotherapy by analyzing patients’ gut microbiota in detail and developing personalized dietary regimens that take into account the individual’s condition.

This review has several limitations. First, the broad applicability of the conclusions is limited by the small sample size of the included studies, which may not be fully representative of all NSCLC patients. Additionally, the included studies were observational and could not clearly establish causality, and larger randomized controlled trials are needed to validate our findings in the future.

## 5 Conclusion

The composition of the gut microbiota is closely associated with the outcomes of immunotherapy in patients with non-small cell lung cancer. Factors such as antibiotics, PPIs, probiotics, and diet can significantly impact the effectiveness of immunotherapy. Our findings provide practical recommendations for the clinical use of gut modulators and dietary strategies.

## Data Availability

Publicly available datasets were analyzed in this study. The datasets used and/or analyzed in this study are available to the corresponding author upon reasonable request.
