# Supplementary material for: Gut microbes and immunotherapy for non-small cell lung cancer: a systematic review
Source: Front Oncol. 2025 May 8;15:1518474. doi: 10.3389/fonc.2025.1518474 (PMC12095033; doi:10.3389/fonc.2025.1518474)
Supplement: Supplementary file 1 [file Table1.docx]

**Appendix 1. Baseline characteristics of included studies**

| First author | Type of study | Country | Sample (n) | M/F | Age (years) | Tumor staging | Treatment type | Sequencing methods | Microbiota |
| --- | --- | --- | --- | --- | --- | --- | --- | --- | --- |
| Li 2024 | Cohort study | China | 39 | 30/9 | 62 ± 8.15 | IIIB-IV | ICI | 16S rRNA | Enhanced ICI efficacy: Clostridia, Lachnospiraceae, Lachnospirales |
| Zhang 2021 | Prospective cohort study | Spain | 69 | 49/20 | 67 | III-IV | ICI | 16S rDNA | Enhanced ICI efficacy: Phascolarctobacterium  Reduced ICI efficacy: Dialister  AE: Bacteroides dorei  NAE: Firmicutes phylum, Bacteroides vulgatus species |
| Grenda 2022 | Cohort study | Poland | 47 | 30/17 | 66(49-79) | IIIB-IV | ICI/ICI+ Chemotherapy | 16S rRNA | Enhanced ICI efficacy: Bacteroidaaceae, Barnesiellaceae, Tannerellaceae  Reduced ICI efficacy: Ruminococcaceae, Clostridia |
| Zhao 2023 | Cohort study | China | 21 | 18/3 | 61.3±9.6 | III-IV | ICI+ Chemotherapy | 16S rRNA | Enhanced ICI efficacy: Bifidobacterium, Escherichia, Sarterella  Reduced ICI efficacy: Lactobacillus, Neisseria |
| Zhang 2021 | Cohort study | China | 75 | 45/30 | / | III-IV | ICI | 16S rRNA | Enhanced ICI efficacy: Alistipes, Anaerostipes, Desulfovibrio, Faecalibacterium, Bifidobacterium  Reduced ICI efficacy: Fusobacterium |
| Dora 2023 | Retrospective cohort study | Hungary | 129 | 53/76 | / | III-IV | ICI | Metagenomic shotgun sequencing | Enhanced ICI efficacy: Bacteroides dorei and Parabacteroides distasonis  Reduced ICI efficacy: Clostridium perfringens, Enterococcus faecium  AE: Lachnospiraceae, Thelephoraceae |
| Fang 2022 | Retrospective cohort study | China | 85 | 54/31 | ＞18 | III-IV | ICI | Metagenomic shotgun sequencing | Enhanced ICI efficacy: Clostridia，Bacteroidia，Bacteroides  Reduced ICI efficacy: Bacteroidia, Bacteroides |
| Jin 2019 | Retrospective cohort study | China | 37 | 29/8 | 62.3 | IIIB-IV | ICI | 16S rRNA | Enhanced ICI efficacy: Alistipes putredinis、Bifidobacterium longum, Prevotella copri  Reduced ICI efficacy: Ruminococcus |
| Sarkar 2023 | Prospective study | USA | 5 | 1/4 | 62(50-68) | III-IV | ICI/ICI+ Chemotherapy | 16S rRNA | Enhanced ICI efficacy: Odoribacter, Gordonibacter, Candidatus Stoquefichus, Escherichia-Shigella, Collinsella, Clostridium sensu stricto 1  Reduced ICI efficacy: Prevotella, Porphyromonas, Streptococcus, Escherichia-Shigella, Akkermansia |
| Hakozaki 2020 | Prospective study | Japan | 70 | / | 70 | IIIB-IV | ICI | 16S rRNA | Enhanced ICI efficacy: Ruminococcaceae UCG 13, Agathobacter  AE: Agathobacter  NAE: Lactobacillaceae and Raoultella, Akkermansia, |
| Shoji 2022 | Prospective observational study | Japan | 28 | 21/7 | 71(56–88) | IIA- IV | ICI | 16S rRNA | Enhanced ICI efficacy: g_Blautia  Reduced ICI efficacy: o_RF32  AE: RF32 unclassified;  NAE: Blautia |
| Dora 2023 | Cohort study | Hungary | 62 | / | / | IIIB-IV | ICI | Metagenomic shotgun sequencing | Enhanced ICI efficacy: Streptococcus salivarius, Streptococcus vestibularis, Bifidobacterium breve  Reduced ICI efficacy: Alistipes shahii, Alistipes finegoldii, Barnesiella visceriola  NAE: Absiella, Blautia |
| Peng 2020 | Prospective study | China | 63 | 53/10 | 61(39–81) | IV | ICI | Illumina HiSeq | Enhanced ICI efficacy: Parabacteroides and Methanobrevibacter  Reduced ICI efficacy: Veillonella, Selenomonadales, Negativicutes |
| He 2021 | Cohort study | China | 16 | / | 18-79 | III-IV | ICI | 16S rRNA | Enhanced ICI efficacy: Escherichia-Shigella, Akkermansia , Olsenella |
| Katayama 2019 | Retrospective study | Japan | 17 | 13/4 | 70(56-83) | III-IV | ICI | 16S rRNA | Enhanced ICI efficacy: Lactobacillus, Clostridium, and Syntrophococcus  Reduced ICI efficacy: Bilophila, Sutterella, Parabacteroides |

**Appendix 2**. **NOS quality scores for included studies**

| **Author** | **Representativeness of the exposed cohort** | **Selection of the non-exposed cohort** | **Ascertainment of exposure** | **Demonstration that outcome of interest was not present at start of study** | **Comparability of cohorts on the basis of the design or analysis** | **Assessment of outcome** | **Was follow-up long enough for outcomes to occur** | **Adequacy of follow up of cohorts** | **Total scores** |
| --- | --- | --- | --- | --- | --- | --- | --- | --- | --- |
| Li 2024 | ★ | ★ | ★ | ★ | ★☆ | ★ | ★ | ☆ | 7 |
| Zhang 2021 | ★ | ★ | ★ | ★ | ★☆ | ★ | ★ | ★ | 8 |
| Grenda 2022 | ★ | ★ | ★ | ★ | ★★ | ★ | ☆ | ☆ | 7 |
| Zhao 2023 | ★ | ★ | ★ | ★ | ★☆ | ★ | ☆ | ☆ | 6 |
| Zhang 2021 | ★ | ★ | ★ | ★ | ★☆ | ★ | ★ | ☆ | 7 |
| Dora 2023 | ★ | ★ | ★ | ★ | ★☆ | ★ | ☆ | ☆ | 6 |
| Fang 2022 | ★ | ★ | ★ | ★ | ★☆ | ★ | ★ | ☆ | 7 |
| Jin 2019 | ★ | ★ | ★ | ★ | ★☆ | ★ | ★ | ★ | 8 |
| Sarkar 2023 | ★ | ★ | ★ | ★ | ★★ | ★ | ☆ | ☆ | 7 |
| Hakozaki 2020 | ★ | ★ | ★ | ★ | ★☆ | ★ | ★ | ★ | 8 |
| Shoji 2022 | ★ | ★ | ★ | ★ | ★★ | ★ | ☆ | ★ | 8 |
| Dora 2023 | ★ | ★ | ★ | ★ | ★☆ | ★ | ★ | ★ | 8 |
| Peng 2020 | ★ | ★ | ★ | ★ | ★☆ | ★ | ☆ | ★ | 7 |
| He 2021 | ★ | ★ | ★ | ★ | ★☆ | ★ | ★ | ☆ | 7 |
| Katayama 2019 | ★ | ★ | ★ | ★ | ★☆ | ★ | ☆ | ★ | 7 |
