# Supplementary material for: Gut microbes and immunotherapy for non-small cell lung cancer: a systematic review
Source: Front Oncol. 2025 May 8;15:1518474. doi: 10.3389/fonc.2025.1518474 (PMC12095033; doi:10.3389/fonc.2025.1518474)
Supplement: Supplementary file 2 [file DataSheet1.pdf]

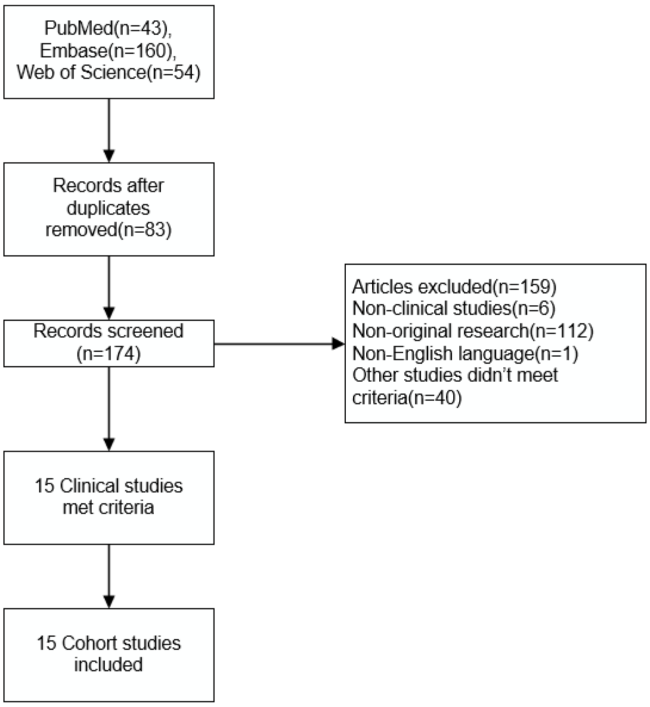

Fig.1 Flowchart of screening for antibiotic exposure

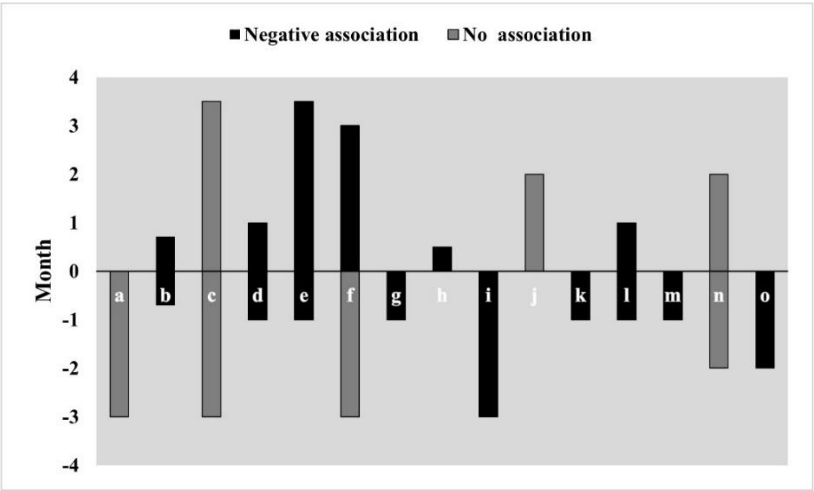

Fig.2 Antibiotic exposure time

a. Zhang, Feiyu et al.,2021; b. Hamada, Kazuyuki et al.,2021; c. Kaderbhai, Coureche et al.,2017; d. Zhao, Sha et al.,2019; e. Ruiz-Patiño, Alejandro et al.,2020; f. Galli, Giulia et al.,2019; g. Lu, Po-Hsien et al.,2021; h. Geum, Min Jung et al.,2021; i. Alkan Şen, Gülin et al.,2023; j.Nyein, Andrew F et al.,2022; k. Hakozaki, Taiki et al.,2020; l. Castello, Angelo et al.,2021; m. Hakozaki, Taiki et al.,2019; n. Martinez-Mugica Barbosa, C et al.,2022; o. Schett, Anne et al.,2020

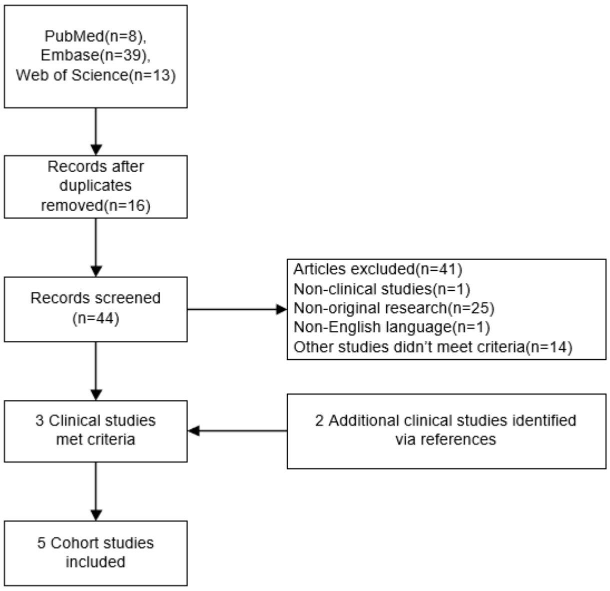

Fig.3 Flowchart of screening for PPI exposure

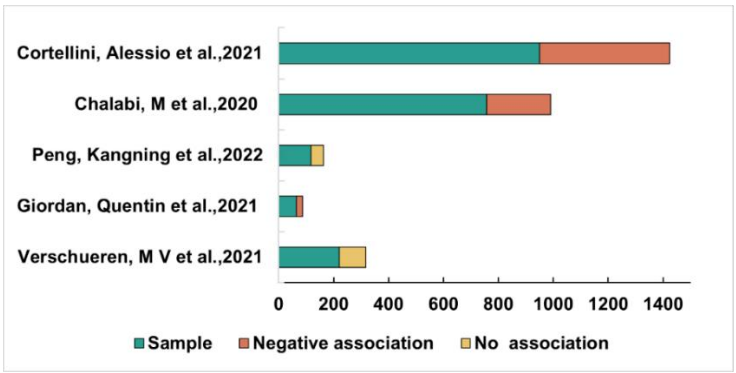

Fig.4 Effect of PPI exposure on the efficacy of ICIs in NSCLC.

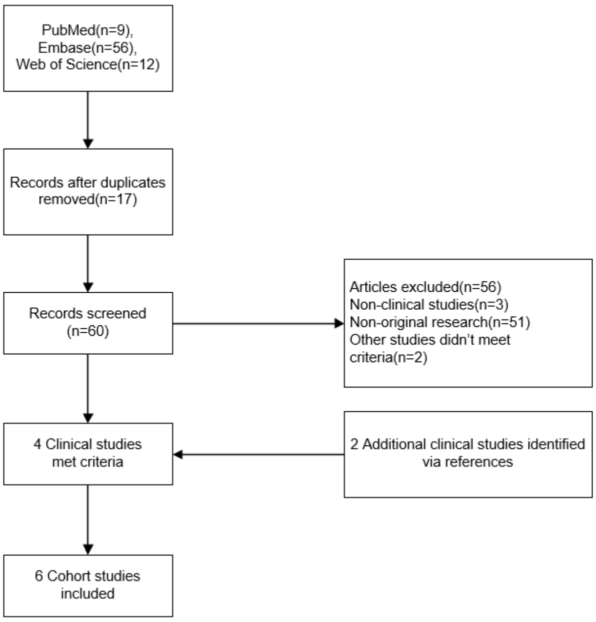

Fig.5 Screening flowchart for probiotic use

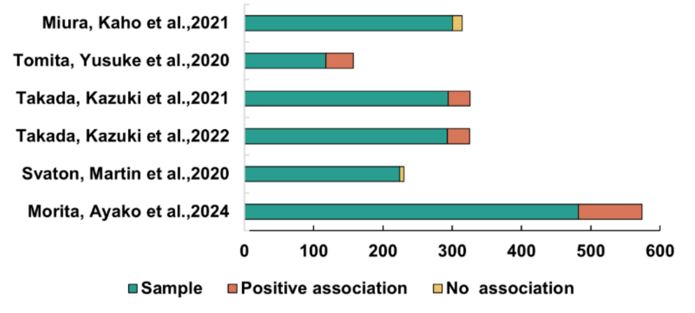

Fig.6 Effect of probiotic application on the efficacy of ICIs in NSCLC.

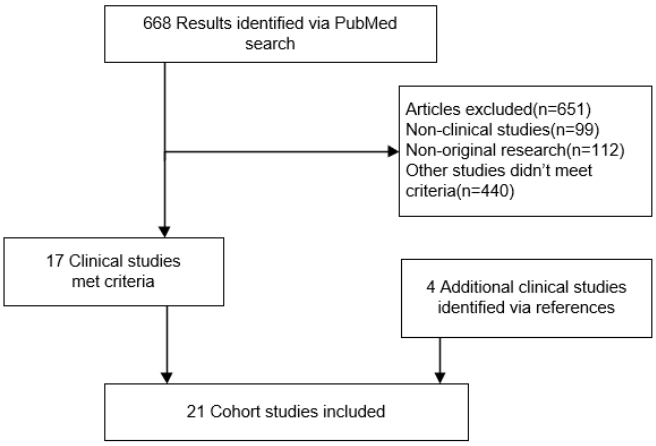

Fig.7 Screening flowchart for dietary intervention
