# Supplementary material for: Gut microbes and immunotherapy for non-small cell lung cancer: a systematic review
Source: Front Oncol. 2025 May 8;15:1518474. doi: 10.3389/fonc.2025.1518474 (PMC12095033; doi:10.3389/fonc.2025.1518474)
Supplement: Supplementary file 3 [file DataSheet2.pdf]

literatures“Therapeutic reactions and adverse effects of ICIs“

serial number	PMID/PUI/ISSN	Year	Property	Classification	Note 1	Note 2
1	33020237	2020	Note	Not-original research		
2	29282371	2017	Editorial Material	Not-original research		
3	29614305	2018	Note	Not-original research		
4	29688263	2018	Editorial Material	Not-original research		
5	31345329	2019	Editorial Material	Not-original research		
6	32016028	2019	Note	Not-original research		
7	36478623	2023	Letter	Not-original research		
8	29509750	2018	Editorial Material	Not-original research		
9	29251088	2017	Editorial	Not-original research		
10	32950294	2021	Editorial Material	Not-original research		
11	35235771	2022	Note	Not-original research		
12	31833336	2019	Editorial Material	Not-original research		
13	31668739	2019	Editorial Material	Not-original research		
14	35584634	2022	Note	Not-original research		
15	29217839	2018	Editorial Material	Not-original research		
16	L620600313	2018	Short Survey	Not-original research		
17	35982190	2022	Article in Press	Not-original research		
18	37690533	2023	Review 1	Not-original research		
19	34521783	2021	Review 2	Not-original research		
20	33123253	2020	Review 3	Not-original research		
21	35860836	2022	Review 4	Not-original research		
22	31003463	2019	Review 5	Not-original research		
23	31192215	2019	Review 6	Not-original research		
24	36103047	2023	Review 7	Not-original research		
25	37823218	2024	Review 8	Not-original research		
26	35469916	2022	Review 9	Not-original research		
27	36279081	2022	Review 10	Not-original research		
28	31450659	2019	Review 11	Not-original research		
29	35741028	2022	Review 12	Not-original research		
30	38132121	2023	Review 13	Not-original research		
31	29780391	2018	Review 14	Not-original research		
32	30122997	2018	Review 15	Not-original research		
33	31575023	2019	Review 16	Not-original research		
34	37675321	2023	Review 17	Not-original research		
35	36969071	2023	Review 18	Not-original research		
36	36776847	2023	Review 19	Not-original research		
37	32676307	2020	Review 20	Not-original research		
38	36382735	2023	Review 21	Not-original research		
39	33145316	2020	Review 22	Not-original research		
40	33346129	2021	Review 23	Not-original research		
41	35223505	2022	Review 24	Not-original research		
42	36911704	2023	Review 25	Not-original research		
43	35449051	2022	Review 26	Not-original research		
44	37634980	2023	Review 27	Not-original research		
45	34539138	2021	Review 28	Not-original research		
46	35636978	2022	Review 29	Not-original research		
47	36513509	2022	Review 30	Not-original research		
48	37222345	2023	Review 31	Not-original research		
49	38241975	2024	Review 32	Not-original research		
50	30497521	2018	Review 33	Not-original research		
51	37441065	2023	Review 34	Not-original research		
52	31663125	2020	Review 35	Not-original research		
53	34638308	2021	Review 36	Not-original research		
54	31827982	2019	Review 37	Not-original research		
55	31884204	2020	Review 38	Not-original research		
56	37999101	2023	Review 39	Not-original research		
57	35841869	2022	Review 40	Not-original research		
58	35982190	2022	Review 41	Not-original research		
59	32100550	2020	Review 42	Not-original research		
60	38361936	2024	Review 43	Not-original research		
61	34229597	2022	Review 44	Not-original research		
62	35847121	2022	Review 45	Not-original research		
63	36358736	2022	Review 46	Not-original research		
64	34638770	2021	Review 47	Not-original research		
65	34444834	2021	Review 48	Not-original research		
66	36798129	2023	Review 49	Not-original research		
67	35252014	2022	Review 50	Not-original research		
68	37365438	2023	Review 51	Not-original research		
69	38074650	2023	Review 52	Not-original research		
70	30232229	2018	Review 53	Not-original research		
71	30936499	2019	Review 54	Not-original research		
72	31827379	2019	Review 55	Not-original research		
73	37947202	2023	Review 56	Not-original research		
74	34992611	2021	Review 57	Not-original research		
75	31723355	2019	Review 58	Not-original research		
76	32121290	2020	Review 59	Not-original research		
77	32542150	2020	Review 60	Not-original research		
78	37076040	2023	Review 61	Not-original research		
79	38451000	2024	Review 62	Not-original research		
80	31616428	2019	Review 63	Not-original research		
81	28889085	2017	Review 64	Not-original research		
82	37880100	2024	Review 65	Not-original research		
83	36455219	2022	Review 66	Not-original research		
84	35470301	2022	Review 67	Not-original research		

85	31896938	2019	Review 68	Not-original research		
86	31597596	2019	Review 69	Not-original research		
87	36805984	2022	Review 70	Not-original research		
88	29029813	2017	Review 71	Not-original research		
89	30393044	2018	Review 72	Not-original research		
90	36428677	2022	Review 73	Not-original research		
91	33989733	2021	Review 74	Not-original research		
92	36230724	2022	Review 75	Not-original research		
93	L2007004684	2020	Review 76	Not-original research		
94	38292482	2024	Review 77	Not-original research		
95	33375062	2020	Review 78	Not-original research		
96	32721966	2021	Review 79	Not-original research		
97	34002348	2021	Review 80	Not-original research		
98	32375310	2020	Review 81	Not-original research		
99	34273520	2022	Review 82	Not-original research		
100	35757563	2020	Review 83	Not-original research		
101	34884327	2021	Review 84	Not-original research		
102	38082435	2023	Review 85	Not-original research		
103	31159348	2019	Review 86	Not-original research		
104	37742728	2023	Review 87	Not-original research		
105	32575876	2020	Review 88	Not-original research		
106	32619934	2020	Review 89	Not-original research		
107	36765735	2023	Review 90	Not-original research		
108	31226812	2019	Review 91	Not-original research		
109	37197624	2023	Review 92	Not-original research		
110	31268133	2019	Review 93	Not-original research		
111	34125407	2021	Review 94	Not-original research		
112	37326100	2023	Review 95	Not-original research		
113	36230554	2022	Review 96	Not-original research		
114	37131214	2023	Review 97	Not-original research		
115	36519027	2022	Review 98	Not-original research		
116	33798103	2021	Review 99	Not-original research		
117	36033445	2022	Review 100	Not-original research		
118	34429006	2022	Review 101	Not-original research		
119	29313208	2018	Review 102	Not-original research		
120	36672796	2022	Review 103	Not-original research		
121	37416062	2023	Review 104	Not-original research		
122	33653420	2021	Review 105	Not-original research		
123	38201531	2023	Review 106	Not-original research		
124	35200543	2022	Review 107	Not-original research		
125	37835378	2023	Review 108	Not-original research		
126	37504362	2023	Review 109	Not-original research		
127	35359844	2022	Review 110	Not-original research		
128	31816940	2019	Review 111	Not-original research		
129	33816289	2021	Review 112	Not-original research		
130	32344837	2020	Review 113	Not-original research		
131	33294085	2020	Review 114	Not-original research		
132	37037418	2023	Review 115	Not-original research		
133	37046762	2023	Review 116	Not-original research		
134	34162429	2021	Review 117	Not-original research		
135	32878769	2020	Review 118	Not-original research		
136	31911189	2020	Review 119	Not-original research		
137	32899961	2020	Review 120	Not-original research		
138	36429112	2022	Review 121	Not-original research		
139	30159703	2018	Review 122	Not-original research		
140	35002732	2021	Review 123	Not-original research		
141	L2021339305	2022	Review 124	Not-original research		
142	30991686	2019	Review 125	Not-original research		
143	33684660	2021	Review 126	Not-original research		
144	36937417	2023	Review 127	Not-original research		
145	33686787	2021	Review 128	Not-original research		
146	37180438	2023	Review 129	Not-original research		
147	31063806	2019	Review 130	Not-original research		
148	29781826	2018	Review 131	Not-original research		
149	35884388	2022	Review 132	Not-original research		
150	33162990	2020	Review 133	Not-original research		
151	36032113	2022	Review 134	Not-original research		
152	35403533	2022	Review 135	Not-original research		
153	34486684	2021	Review 136	Not-original research		
154	31741763	2019	Review 137	Not-original research		
155	38042137	2024	Review 138	Not-original research		
156	33875382	2021	Review 139	Not-original research		
157	35603657	2022	Review 140	Not-original research		
158	34174268	2021	Review 141	Not-original research		
159	36110546	2022	Review 142	Not-original research		
160	38103006	2023	Review 143	Not-original research		
161	35123155	2022	Review 144	Not-original research		
162	36090978	2022	Review 145	Not-original research		
163	38571678	2024	Review 146	Not-original research		
164	36982303	2023	Review 147	Not-original research		
165	35397955	2022	Review 148	Not-original research		
166	35770869	2023	Review 149	Not-original research		
167	L2025678359	2023	Review 150	Not-original research		
168	37950236	2023	Review 151	Not-original research		
169	30530851	2019	Review 152	Not-original research		
170	34400407	2021	Review 153	Not-original research		

171	33227982	2020	Review 154	Not-original research		
172	35672823	2021	Review 155	Not-original research		
173	30864326	2019	Review 156	Not-original research		
174	L2008063006	2021	Review 157	Not-original research		
175	36774235	2023	Clinical study	Other studies didn't meet criteria		
176	34593446	2021	Clinical study	Other studies didn't meet criteria		
177	34400292	2021	Clinical study	Other studies didn't meet criteria		
178	30885328	2019	Clinical study	Other studies didn't meet criteria		
179	32705787	2020	Clinical study	Other studies didn't meet criteria		
180	35676015	2022	Clinical study	Other studies didn't meet criteria		
181	37442801	2023	Clinical study	Other studies didn't meet criteria		
182	35267634	2022	Clinical study	Other studies didn't meet criteria		
183	31097097	2019	Clinical study	Other studies didn't meet criteria		
184	34857829	2021	Clinical study	Other studies didn't meet criteria		
185	33182025	2020	Clinical study	Other studies didn't meet criteria		
186	35986342	2022	Clinical study	Other studies didn't meet criteria		
187	38010059	2023	Clinical study	Other studies didn't meet criteria		
188	38201474	2023	Clinical study	Other studies didn't meet criteria		
189	33332621	2021	Clinical study	Other studies didn't meet criteria		
190	34066877	2021	Clinical study	Other studies didn't meet criteria		
191	37160893	2023	Clinical study	Other studies didn't meet criteria		
192	36640544	2023	Clinical study	Other studies didn't meet criteria		
193	33720422	2021	Clinical study	Other studies didn't meet criteria		
194	35655708	2022	Clinical study	Other studies didn't meet criteria		
195	32900789	2020	Clinical study	Other studies didn't meet criteria		
196	36143126	2022	Clinical study	Other studies didn't meet criteria		
197	31292268	2020	Clinical study	Other studies didn't meet criteria		
198	37597304	2023	Clinical study	Other studies didn't meet criteria		
199	a PhD thesis	2019	Clinical study	Other studies didn't meet criteria		
200	31718585	2019	Clinical study	Other studies didn't meet criteria		
201	33212418	2021	Clinical study	Other studies didn't meet criteria		
202	37937583	2023	Clinical study	Other studies didn't meet criteria		
203	35073876	2022	Clinical study	Other studies didn't meet criteria		
204	27717798	2016	Clinical study	Other studies didn't meet criteria		
205	36358819	2022	Clinical study	Other studies didn't meet criteria		
206	32855157	2020	Clinical study	Other studies didn't meet criteria		
207	36387171	2022	Clinical study	Other studies didn't meet criteria		
208	37662409	2023	Clinical study	Other studies didn't meet criteria		
209	35543371	2022	Clinical study	Other studies didn't meet criteria		
210	31919759	2020	Clinical study	Other studies didn't meet criteria		
211	34777340	2021	Clinical study	Other studies didn't meet criteria		
212	33465728	2021	Clinical study	Other studies didn't meet criteria		
213	30666802	2019	Clinical study	Other studies didn't meet criteria		
214	30854072	2019	Clinical study	Other studies didn't meet criteria		
215	34282636	2022	Clinical study	Other studies didn't meet criteria		
216	37737046	2023	Clinical study	Other studies didn't meet criteria		
217	36164854	2022	Clinical study	Other studies didn't meet criteria		
218	33826550	2021	Clinical study	Other studies didn't meet criteria		
219	38196128	2024	Clinical study	Other studies didn't meet criteria		
220	33154150	2020	Clinical study	Other studies didn't meet criteria		
221	37601658	2023	Clinical study	Other studies didn't meet criteria		
222	33177060	2021	Clinical study	Other studies didn't meet criteria		
223	37944495	2023	Clinical study	Other studies didn't meet criteria		
224	33329547	2020	Clinical study	Other studies didn't meet criteria		
225	29617710	2018	Clinical study	Other studies didn't meet criteria		
226	34413300	2021	Clinical study	Other studies didn't meet criteria		
227	a PhD thesis	2022	Clinical study	Other studies didn't meet criteria		
228	37286305	2023	Clinical study	Other studies didn't meet criteria		
229	33128571	2021	Clinical study	Other studies didn't meet criteria		
230	31745593	2020	Clinical study	Other studies didn't meet criteria		
231	35958344	2022	Clinical study	Other studies didn't meet criteria		
232	34247166	2021	Clinical study	Other studies didn't meet criteria		
233	31292005	2019	Clinical study	Other studies didn't meet criteria		
234	33158018	2020	Clinical study	Other studies didn't meet criteria		
235	37207204	2023	Clinical study	Other studies didn't meet criteria		
236	33387437	2023	Clinical study	Other studies didn't meet criteria		
237	37114123	2023	Clinical study	Other studies didn't meet criteria		
238	37444378	2023	Clinical study	Other studies didn't meet criteria		
239	35115705	2022	Clinical study	Other studies didn't meet criteria		
240	34912592	2021	Clinical study	Other studies didn't meet criteria		
241	35073853	2022	Clinical study	Other studies didn't meet criteria		
242	38103007	2023	Clinical study	Other studies didn't meet criteria		
243	36063818	2022	Clinical study	Other studies didn't meet criteria		
244	32014010	2020	Clinical study	Other studies didn't meet criteria		
245	35792976	2022	Clinical study	Other studies didn't meet criteria		
246	L2030948453	2024	Conference Abstract	Not-original research		
247	L2021328735	2022	Conference Abstract	Not-original research		
248	L2020168369	2022	Conference Abstract	Not-original research		
249	L2020177120	2022	Conference Abstract	Not-original research		
250	L2027892560	2023	Conference Abstract	Not-original research		
251	L2027889219	2023	Conference Abstract	Not-original research		
252	L635023968	2021	Conference Abstract	Not-original research		
253	L635024046	2021	Conference Abstract	Not-original research		
254	1556-0864	2019	Conference Abstract	Not-original research		
255	L638834929	2022	Conference Abstract	Not-original research		
256	L638834487	2022	Conference Abstract	Not-original research		

257	L625970375	2019	Conference Abstract	Not-original research		
258	37785836	2023	Conference Abstract	Not-original research		
259	L635588931	2021	Conference Abstract	Not-original research		
260	L635024071	2021	Conference Abstract	Not-original research		
261	L2020176110	2022	Conference Abstract	Not-original research		
262	0923-7534	2019	Conference Abstract	Not-original research		
263	L632807424	2020	Conference Abstract	Not-original research		
264	38385162	2024	Fundamental research	Non-clinical studies		
265	37742282	2023	Fundamental research	Non-clinical studies		
266	34253574	2022	Fundamental research	Non-clinical studies		
267	32944086	2020	Fundamental research	Non-clinical studies		
268	37635362	2023	Fundamental research	Non-clinical studies		
269	37289890	2023	Fundamental research	Non-clinical studies		
270	34006584	2022	Fundamental research	Non-clinical studies		
271	35346337	2022	Fundamental research	Non-clinical studies		
272	35217892	2022	Fundamental research	Non-clinical studies		
273	38415012	2024	Selected article	Cohort studies included	Fig.2 h	
274	34063829	2021	Selected article	Cohort studies included	Fig.2 a,Fig.3 a	Immune-Related Adverse Events
275	36551735	2022	Selected article	Cohort studies included	Fig.2 b	
276	36205311	2023	Selected article	Cohort studies included	Fig.2 c	
277	36358821	2022	Clinical study	Other studies didn't meet criteria		
278	34028936	2021	Selected article	Cohort studies included	Fig.2 d	
279	37894458	2023	Selected article	Cohort studies included	Fig.2 e,Fig.3 e	Immune-Related Adverse Events
280	35530307	2022	Selected article	Cohort studies included	Fig.2 f	
281	31026576	2019	Selected article	Cohort studies included	Fig.2 g	
282	37350807	2023	Selected article	Cohort studies included	Fig.2 i	
283	32847937	2020	Selected article	Cohort studies included	Fig.2 j,Fig.3 j	Immune-Related Adverse Events
284	36353732	2022	Selected article	Cohort studies included	Fig.2 k,Fig.3 k	Immune-Related Adverse Events
285	37197440	2023	Selected article	Cohort studies included	Fig.2 l,Fig.3 l	Immune-Related Adverse Events
286	32329229	2020	Selected article	Cohort studies included	Fig.2 m	
287	34664203	2021	Selected article	Cohort studies included	Fig.2 n	
288	32010563	2019	Selected article	Cohort studies included	Fig.2 o	
289	L625967438	2019	Conference Abstract	Not-original research		
290	L625970218	2019	Conference Abstract	Not-original research		
291	L633439845	2020	Conference Abstract	Not-original research		
292	L631546867	2020	Conference Abstract	Not-original research		
293	L624231174	2018	Conference Abstract	Not-original research		
294	L2023625746	2023	Conference Abstract	Not-original research		
295	L635586728	2021	Conference Abstract	Not-original research		
296	L641664711	2023	Conference Abstract	Not-original research		
297	L2007890825	2020	Conference Abstract	Not-original research		
298	L635307037	2021	Conference Abstract	Not-original research		
299	L628559638	2019	Conference Abstract	Not-original research		
300	L628089661	2019	Conference Abstract	Not-original research		
301	L635904286	2021	Conference Abstract	Not-original research		
302	L2028084980	2023	Conference Abstract	Not-original research		
303	L2020097333	2022	Conference Abstract	Not-original research		
304	L2020097384	2022	Conference Abstract	Not-original research		
305	L2028085324	2023	Conference Abstract	Not-original research		
306	L2015167818	2021	Conference Abstract	Not-original research		
307	L2015163488	2021	Conference Abstract	Not-original research		
308	L633629968	2020	Conference Abstract	Not-original research		
309	0732-183X	2020	Conference Abstract	Not-original research		
310	L638843065	2022	Conference Abstract	Not-original research		
311	L629438818	2019	Conference Abstract	Not-original research		
312	L2022887218	2023	Conference Abstract	Not-original research		
313	L635024063	2020	Conference Abstract	Not-original research		
314	0008-5472	2020	Conference Abstract	Not-original research		
315	L635590526	2021	Conference Abstract	Not-original research		
316	L634192418	2020	Conference Abstract	Not-original research		
317	L636558000	2021	Conference Abstract	Not-original research		
318	L636558014	2021	Conference Abstract	Not-original research		
319	0016-5085	2019	Conference Abstract	Not-original research		
320	L2003406354	2019	Conference Abstract	Not-original research		
321	L635024003	2020	Conference Abstract	Not-original research		
322	L2030947185	2024	Conference Abstract	Not-original research		
323	L637180633	2022	Conference Abstract	Not-original research		
324	L639737852	2022	Conference Abstract	Not-original research		
325	L632365785	2020	Conference Abstract	Not-original research		
326	L2001207014	2018	Conference Abstract	Not-original research		
327	L635612422	2021	Conference Abstract	Not-original research		
328	L635905172	2021	Conference Abstract	Not-original research		
329	L2003406779	2019	Conference Abstract	Not-original research		
330	L2020098010	2022	Conference Abstract	Not-original research		
331	L2003405809	2019	Conference Abstract	Not-original research		
332	L2011421808	2021	Conference Abstract	Not-original research		
333	L2015170175	2021	Conference Abstract	Not-original research		
334	L2011421824	2021	Conference Abstract	Not-original research		
335	L638834494	2022	Conference Abstract	Not-original research		
336	L635025172	2020	Conference Abstract	Not-original research		
337	0732-183X	2020	Conference Abstract	Not-original research		
338	L639737599	2022	Conference Abstract	Not-original research		
339	L635023994	2020	Conference Abstract	Not-original research		
340	L628119000	2019	Conference Abstract	Not-original research		
341	L629324364	2019	Conference Abstract	Not-original research		
342	L625970017	2018	Conference Abstract	Not-original research		

343	L626627085	2018	Conference Abstract	Not-original research		
344	L641472914	2023	Conference Abstract	Not-original research		
345	L643160287	2023	Conference Abstract	Not-original research		
346	L635024077	2020	Conference Abstract	Not-original research		
347	L638834687	2022	Conference Abstract	Not-original research		
348	L2019358151	2022	Conference Abstract	Not-original research		
349	L636986417	2021	Conference Abstract	Not-original research		
350	0008-5472	2022	Conference Abstract	Not-original research		
351	L635023972	2020	Conference Abstract	Not-original research		

literatures"Antibiotic exposures"

serial number	PMID/PUI	Year	Property	Classification	Note	
1	34063829	2021	Selected article	Cohort studies included	Supplementary Figure 2.a	
2	34593446	2021	Selected article	Cohort studies included	Supplementary Figure 2.b	
3	28551664	2017	Selected article	Cohort studies included	Supplementary Figure 2.c	
4	30885328	2019	Selected article	Cohort studies included	Supplementary Figure 2.d	
5	32705787	2020	Selected article	Cohort studies included	Supplementary Figure 2.e	
6	31097097	2019	Selected article	Cohort studies included	Supplementary Figure 2.f	
7	33332621	2021	Selected article	Cohort studies included	Supplementary Figure 2.g	
8	34066877	2021	Selected article	Cohort studies included	Supplementary Figure 2.h	
9	37597304	2023	Selected article	Cohort studies included	Supplementary Figure 2.i	
10	35073876	2022	Selected article	Cohort studies included	Supplementary Figure 2.j	
11	32847937	2020	Selected article	Cohort studies included	Supplementary Figure 2.k	
12	33803006	2021	Selected article	Cohort studies included	Supplementary Figure 2.l	
13	30854072	2019	Selected article	Cohort studies included	Supplementary Figure 2.m	
14	36164854	2022	Selected article	Cohort studies included	Supplementary Figure 2.n	
15	31745593	2020	Selected article	Cohort studies included	Supplementary Figure 2.o	
16	33934059	2021	Clinical study	Other studies didn't meet criteria		
17	33212418	2021	Clinical study	Other studies didn't meet criteria		
18	36640544	2023	Clinical study	Other studies didn't meet criteria		
19	35860836	2022	Clinical study	Other studies didn't meet criteria		
20	34400292	2021	Clinical study	Other studies didn't meet criteria		
21	33154150	2020	Clinical study	Other studies didn't meet criteria		
22	33128571	2021	Clinical study	Other studies didn't meet criteria		
23	29617710	2018	Clinical study	Other studies didn't meet criteria		
24	35115705	2022	Clinical study	Other studies didn't meet criteria		
25	37197440	2023	Clinical study	Other studies didn't meet criteria		
26	37197440	2023	Clinical study	Other studies didn't meet criteria		
27	34777340	2021	Clinical study	Other studies didn't meet criteria		
28	35792976	2022	Clinical study	Other studies didn't meet criteria		
29	36551735	2022	Clinical study	Other studies didn't meet criteria		
30	31919759	2020	Clinical study	Other studies didn't meet criteria		
31	32010563	2019	Clinical study	Other studies didn't meet criteria		
32	33826550	2021	Clinical study	Other studies didn't meet criteria		
33	31718585	2019	Clinical study	Other studies didn't meet criteria		
34	32900789	2020	Clinical study	Other studies didn't meet criteria		
35	35389889	2022	Clinical study	Other studies didn't meet criteria		
36	31292005	2019	Clinical study	Other studies didn't meet criteria		
37	34282636	2022	Clinical study	Other studies didn't meet criteria		
38	32855157	2020	Clinical study	Other studies didn't meet criteria		
39	34242975	2021	Clinical study	Other studies didn't meet criteria		
40	37160893	2023	Clinical study	Other studies didn't meet criteria		
41	32329229	2020	Clinical study	Other studies didn't meet criteria		
42	35780526	2022	Clinical study	Other studies didn't meet criteria		
43	33720422	2021	Clinical study	Other studies didn't meet criteria		
44	35543371	2022	Clinical study	Other studies didn't meet criteria		
45	31292268	2020	Clinical study	Other studies didn't meet criteria		
46	35655708	2022	Clinical study	Other studies didn't meet criteria		
47	35655708	2022	Clinical study	Other studies didn't meet criteria		
48	38201474	2023	Clinical study	Other studies didn't meet criteria		
49	34857829	2021	Clinical study	Other studies didn't meet criteria		
50	36774235	2023	Clinical study	Other studies didn't meet criteria		
51	35267634	2022	Clinical study	Other studies didn't meet criteria		
52	36072895	2022	Clinical study	Other studies didn't meet criteria		
53	33465728	2021	Clinical study	Other studies didn't meet criteria		
54	34028936	2021	Clinical study	Other studies didn't meet criteria		
55	38010059	2024	Clinical study	Other studies didn't meet criteria		
56	27717798	2016	Fundamental research	Non-clinical studies		
57	35530307	2022	Fundamental research	Non-clinical studies		
58	37289890	2023	Fundamental research	Non-clinical studies		
59	35346337	2022	Fundamental research	Non-clinical studies		
60	35115521	2022	Fundamental research	Non-clinical studies		
61	34253574	2022	Fundamental research	Non-clinical studies		
62	33798103	2021	/	/		
63	L635024046	2020	Conference Abstract	Not-original research		
64	L2027892588	2023	Conference Abstract	Not-original research		
65	L636558000	2021	Conference Abstract	Not-original research		
66	L635023972	2020	Conference Abstract	Not-original research		
67	L2000708649	2018	Note	Not-original research		
68	L2017598818	2022	Short Survey	Not-original research		
69	L628089661	2018	Conference Abstract	Not-original research		
70	L2015169378	2021	Conference Abstract	Not-original research		

71	L641664711	2023	Conference Abstract	Not-original research		
72	L635590526	2021	Conference Abstract	Not-original research		
73	36902013	2023	A Case Report	Not-original research		
74	L636986417	2021	Conference Abstract	Not-original research		
75	L636605883	2020	Conference Abstract	Not-original research		
76	L635904286	2021	Conference Abstract	Not-original research		
77	L625970017	2018	Conference Abstract	Not-original research		
78	L2010099538	2020	Letter	Not-original research		
79	L625970375	2018	Conference Abstract	Not-original research		
80	L628559638	2018	Conference Abstract	Not-original research		
81	L2023626754	2023	Conference Abstract	Not-original research		
82	L2015167818	2021	Conference Abstract	Not-original research		
83	L636558014	2021	Conference Abstract	Not-original research		
84	L632809151	2020	Conference Abstract	Not-original research		
85	L2003405809	2019	Conference Abstract	Not-original research		
86	L620503943	2018	Short Survey	Not-original research		
87	L2014470451	2021	Letter	Not-original research		
88	L629904758	2019	Conference Abstract	Not-original research		
89	L623320335	2018	Editorial	Not-original research		
90	L2028084980	2023	Conference Abstract	Not-original research		
91	L622519741	2018	Editorial	Not-original research		
92	L634192418	2020	Conference Abstract	Not-original research		
93	L630808547	2019	Note	Not-original research		
94	L633250669	2020	Conference Abstract	Not-original research		
95	L2020176110	2022	Conference Abstract	Not-original research		
96	L637180633	2022	Conference Abstract	Not-original research		
97	L2003406779	2019	Conference Abstract	Not-original research		
98	L618366968	2017	Editorial	Not-original research		
99	L628119000	2019	Conference Abstract	Not-original research		
100	L2015163488	2021	Conference Abstract	Not-original research		
101	L635024077	2020	Conference Abstract	Not-original research		
102	L635024003	2020	Conference Abstract	Not-original research		
103	L635586728	2021	Conference Abstract	Not-original research		
104	L2016112291	2022	Conference Abstract	Not-original research		
105	L2020177120	2022	Conference Abstract	Not-original research		
106	L625970218	2018	Conference Abstract	Not-original research		
107	L629324364	2019	Conference Abstract	Not-original research		
108	L2007890825	2020	Conference Abstract	Not-original research		
109	L2028085324	2023	Conference Abstract	Not-original research		
110	L638843065	2022	Conference Abstract	Not-original research		
111	L2001208088	2018	Conference Abstract	Not-original research		
112	31827982	2019	Review 1	Not-original research		
113	32575876	2020	Review 2	Not-original research		
114	32899961	2020	Review 3	Not-original research		
115	33375194	2020	Review 4	Not-original research		
116	35876944	2022	Review 5	Not-original research		
117	37722262	2023	Review 6	Not-original research		
118	36672796	2022	Review 7	Not-original research		
119	36937417	2023	Review 8	Not-original research		
120	34884327	2021	Review 9	Not-original research		
121	34400407	2021	Review 10	Not-original research		
122	37999101	2023	Review 11	Not-original research		
123	31268133	2019	Review 12	Not-original research		
124	33162990	2020	Review 13	Not-original research		
125	31836241	2020	Review 14	Not-original research		
126	33790679	2021	Review 15	Not-original research		
127	32100550	2020	Review 16	Not-original research		
128	32878769	2020	Review 17	Not-original research		
129	34162429	2021	Review 18	Not-original research		
130	36358736	2022	Review 19	Not-original research		
131	33816289	2021	Review 20	Not-original research		
132	31741763	2019	Review 21	Not-original research		
133	31911189	2020	Review 22	Not-original research		
134	35223505	2022	Review 23	Not-original research		
135	38082435	2023	Review 24	Not-original research		
136	29691222	2018	Review 25	Not-original research		
137	36765735	2023	Review 26	Not-original research		
138	35841869	2022	Review 27	Not-original research		
139	36776847	2023	Review 28	Not-original research		
140	32173463	2020	Review 29	Not-original research		
141	33770330	2022	Review 30	Not-original research		
142	32944086	2020	Review 31	Not-original research		
143	36291904	2022	Review 32	Not-original research		
144	29313208	2018	Review 33	Not-original research		
145	31884204	2020	Review 34	Not-original research		
146	36805984	2022	Review 35	Not-original research		
147	31896938	2019	Review 36	Not-original research		
148	32179179	2020	Review 37	Not-original research		
149	29509750	2018	Review 38	Not-original research		
150	34832953	2021	Review 39	Not-original research		
151	37197624	2023	Review 40	Not-original research		
152	31597596	2019	Review 41	Not-original research		
153	36911704	2023	Review 42	Not-original research		
154	33819567	2022	Review 43	Not-original research		
155	34486684	2021	Review 44	Not-original research		
156	32375706	2020	Review 45	Not-original research		

157	38103006	2023	Review 46	Not-original research		
158	29781826	2018	Review 47	Not-original research		
159	34992611	2021	Review 48	Not-original research		
160	37365438	2023	Review 49	Not-original research		
161	33771672	2021	Review 50	Not-original research		
162	37189417	2023	Review 51	Not-original research		
163	33123253	2020	Review 52	Not-original research		
164	35449051	2022	Review 53	Not-original research		
165	36969071	2023	Review 54	Not-original research		
166	31003463	2019	Review 55	Not-original research		
167	38361936	2024	Review 56	Not-original research		
168	37441065	2023	Review 57	Not-original research		
169	36090978	2022	Review 58	Not-original research		
170	36382735	2023	Review 59	Not-original research		
171	38074650	2023	Review 60	Not-original research		
172	34125407	2021	Review 61	Not-original research		
173	37690533	2023	Review 62	Not-original research		
174	35967438	2022	Review 63	Not-original research		

literatures"PPI exposures"

serial number	PMID/PUI	Year	Property	Classification	Note1	Note2
1	34857829	2021	Selected article	Cohort studies included		
2	34777340	2021	Selected article	Cohort studies included		
3	34282636	2022	Selected article	Cohort studies included		
4	32115349	2020	Selected article	Cohort studies included	reference (the literature)	
5	33827906	2021	Selected article	Cohort studies included	reference (the literature)	
6	34486684	2021	Review 1	Not-original research		
7	33182025	2020	Review 2	Not-original research		
8	35860836	2022	Review 3	Not-original research		
9	36776847	2023	Review 4	Not-original research		
10	36090978	2022	Review 5	Not-original research		
11	38074650	2023	Review 6	Not-original research		
12	37197624	2023	Review 7	Not-original research		
13	37722262	2023	Review 8	Not-original research		
14	31268133	2019	Review 9	Not-original research		
15	38201531	2023	Review 10	Not-original research		
16	37835378	2023	Review 11	Not-original research		
17	35359844	2022	Review 12	Not-original research		
18	33790679	2021	Review 13	Not-original research		
19	35884388	2022	Review 14	Not-original research		
20	33162990	2020	Review 15	Not-original research		
21	31919759	2020	Clinical study	Other studies didn't meet criteria		
22	37937583	2023	Clinical study	Other studies didn't meet criteria		
23	37737046	2023	Clinical study	Other studies didn't meet criteria		
24	32375706	2020	Clinical study	Other studies didn't meet criteria		
25	33154150	2020	Clinical study	Other studies didn't meet criteria		
26	34593446	2021	Clinical study	Other studies didn't meet criteria		
27	35780526	2022	Clinical study	Other studies didn't meet criteria		
28	30885328	2019	Clinical study	Other studies didn't meet criteria		
29	35655708	2022	Clinical study	Other studies didn't meet criteria		
30	30854072	2019	Clinical study	Other studies didn't meet criteria		
31	37197440	2023	Clinical study	Other studies didn't meet criteria		
32	33934059	2021	Clinical study	Other studies didn't meet criteria		
33	37114123	2023	Clinical study	Other studies didn't meet criteria		
34	33212418	2021	Clinical study	Other studies didn't meet criteria		
35	38201474	2023	Fundamental research	Non-clinical studies		
36	L2026580895	2023	Conference Abstract	Not-original research		
37	L635588931	2021	Conference Abstract	Not-original research		
38	L633027389	2020	Note	Not-original research		
39	L2023625746	2023	Conference Abstract	Not-original research		
40	L641664711	2023	Conference Abstract	Not-original research		
41	L628089661	2018	Conference Abstract	Not-original research		
42	L2028085324	2023	Conference Abstract	Not-original research		
43	L2014470451	2021	Letter	Not-original research		
44	L2015170223	2021	Conference Abstract	Not-original research		
45	L629324364	2019	Conference Abstract	Not-original research		
46	L2025678359	2022	/	/		

literatures"Probiotics applications"

serial number	PMID /PUI	Year	Property	Classification	Note	
1	32575876	2020	Review 1	Not-original research		
2	31226812	2019	Review 2	Not-original research		
3	31159348	2019	Review 3	Not-original research		
4	38042137	2024	Review 4	Not-original research		
5	33801815	2021	Review 5	Not-original research		
6	37742728	2023	Review 6	Not-original research		
7	36672796	2022	Review 7	Not-original research		
8	36230724	2022	Review 8	Not-original research		

9	37999101	2023	Review 9	Not-original research		
10	31268133	2019	Review 10	Not-original research		
11	36428677	2022	Review 11	Not-original research		
12	32878769	2020	Review 12	Not-original research		
13	35469916	2022	Review 13	Not-original research		
14	37634980	2023	Review 14	Not-original research		
15	31911189	2020	Review 15	Not-original research		
16	37947202	2023	Review 16	Not-original research		
17	38717360	2024	Review 17	Not-original research		
18	38082435	2023	Review 18	Not-original research		
19	29691222	2018	Review 19	Not-original research		
20	34273520	2022	Review 20	Not-original research		
21	37046762	2023	Review 21	Not-original research		
22	35841869	2022	Review 22	Not-original research		
23	35234272	2022	Review 23	Not-original research		
24	33770330	2022	Review 24	Not-original research		
25	35123155	2022	Review 25	Not-original research		
26	32944086	2020	Review 26	Not-original research		
27	32375310	2020	Review 27	Not-original research		
28	36805984	2022	Review 28	Not-original research		
29	37205627	2023	Review 29	Not-original research		
30	36429112	2022	Review 30	Not-original research		
31	35924468	2022	Review 31	Not-original research		
32	37197624	2023	Review 32	Not-original research		
33	36911704	2023	Review 33	Not-original research		
34	34486684	2021	Review 34	Not-original research		
35	35757563	2020	Review 35	Not-original research		
36	36519027	2022	Review 36	Not-original research		
37	38490281	2024	Review 37	Not-original research		
38	35770869	2023	Review 38	Not-original research		
39	33123253	2020	Review 39	Not-original research		
40	36798129	2023	Review 40	Not-original research		
41	35884388	2022	Review 41	Not-original research		
42	38074650	2023	Review 42	Not-original research		
43	36110546	2022	Review 43	Not-original research		
44	37690533	2023	Review 44	Not-original research		
45	31827379	2019	Review 45	Not-original research		
46	35252014	2022	Review 46	Not-original research		
47	34393006	2021	Letter	Not-original research		
48	34373189	2021	Letter	Not-original research		
49	31836241	2020	editorial	Not-original research		
50	L2028085324	2023	Conference Abstract	Not-original research		
51	35235771	2022	Comment	Not-original research		
52	34593446	2021	Clinical study	Other studies didn't meet criteria		
53	30864326	2019	Clinical study	Other studies didn't meet criteria		
54	34006584	2022	Fundamental research	Non-clinical studies		
55	33680705	2021	Fundamental research	Non-clinical studies		
56	23354042	2013	Fundamental research	Non-clinical studies		
57	38196128	2024	Selected article	Cohort studies included		
58	32234916	2020	Selected article	Cohort studies included		
59	35780526	2022	Selected article	Cohort studies included		
60	33720422	2021	Selected article	Cohort studies included		
61	32665261	2020	Selected article	Cohort studies included	reference (the literature)	
62	33990133	2021	Selected article	Cohort studies included	reference (the literature)	

literatures"Dietary effects"

serial number	PMID	Year	Property	Classification	Note1	Note2
1	30638420	2019	Selected article	Cohort studies included		
2	29509315	2019	Selected article	Cohort studies included		
3	34836220	2021	Selected article	Cohort studies included		
4	30231542	2018	Selected article	Cohort studies included		
5	31108510	2019	Selected article	Cohort studies included		
6	34385825	2021	Selected article	Cohort studies included		
7	30317146	2019	Selected article	Cohort studies included		
8	28296348	2017	Selected article	Cohort studies included		
9	25519526	2015	Selected article	Cohort studies included		
10	29373513	2018	Selected article	Cohort studies included		
11	31776537	2019	Selected article	Cohort studies included		
12	28526852	2017	Selected article	Cohort studies included		
13	27676396	2016	Selected article	Cohort studies included		
14	25690418	2015	Selected article	Cohort studies included		
15	28213610	2017	Selected article	Cohort studies included		
16	29726951	2018	Selected article	Cohort studies included		
17	29470389	2018	Selected article	Cohort studies included		
18	24336217	2014	Selected article	Cohort studies included	reference (the literature)	
19	23038174	2013	Selected article	Cohort studies included	reference (the literature)	
20	25299601	2014	Selected article	Cohort studies included	reference (the literature)	
21	22649263	2012	Selected article	Cohort studies included	reference (the literature)	
22	28012242	2017	Clinical study	Other studies didn't meet criteria		
23	35114943	2022	Clinical study	Other studies didn't meet criteria		
24	30396006	2018	Clinical study	Other studies didn't meet criteria		
25	32537376	2020	Clinical study	Other studies didn't meet criteria		

26	33075782	2020	Clinical study	Other studies didn't meet criteria		
27	33182700	2020	Clinical study	Other studies didn't meet criteria		
28	25988339	2015	Clinical study	Other studies didn't meet criteria		
29	26235304	2015	Clinical study	Other studies didn't meet criteria		
30	24233256	2015	Clinical study	Other studies didn't meet criteria		
31	33727392	2021	Clinical study	Other studies didn't meet criteria		
32	32330749	2020	Clinical study	Other studies didn't meet criteria		
33	27102333	2016	Clinical study	Other studies didn't meet criteria		
34	35565679	2022	Clinical study	Other studies didn't meet criteria		
35	30425247	2018	Clinical study	Other studies didn't meet criteria		
36	34205926	2021	Clinical study	Other studies didn't meet criteria		
37	31258108	2019	Clinical study	Other studies didn't meet criteria		
38	28179226	2017	Clinical study	Other studies didn't meet criteria		
39	30876827	2020	Clinical study	Other studies didn't meet criteria		
40	28954842	2017	Clinical study	Other studies didn't meet criteria		
41	26987626	2016	Clinical study	Other studies didn't meet criteria		
42	26773784	2016	Clinical study	Other studies didn't meet criteria		
43	36913444	2023	Clinical study	Other studies didn't meet criteria		
44	30845997	2019	Clinical study	Other studies didn't meet criteria		
45	34444797	2021	Clinical study	Other studies didn't meet criteria		
46	34117375	2022	Clinical study	Other studies didn't meet criteria		
47	26228065	2015	Clinical study	Other studies didn't meet criteria		
48	36921804	2023	Clinical study	Other studies didn't meet criteria		
49	30782617	2019	Clinical study	Other studies didn't meet criteria		
50	33809130	2021	Clinical study	Other studies didn't meet criteria		
51	31051503	2019	Clinical study	Other studies didn't meet criteria		
52	33198235	2020	Clinical study	Other studies didn't meet criteria		
53	34020448	2021	Clinical study	Other studies didn't meet criteria		
54	33450667	2021	Clinical study	Other studies didn't meet criteria		
55	37513272	2023	Clinical study	Other studies didn't meet criteria		
56	38408729	2024	Clinical study	Other studies didn't meet criteria		
57	37587110	2023	Clinical study	Other studies didn't meet criteria		
58	32083496	2020	Clinical study	Other studies didn't meet criteria		
59	30269035	2018	Clinical study	Other studies didn't meet criteria		
60	37293209	2023	Clinical study	Other studies didn't meet criteria		
61	33429936	2021	Clinical study	Other studies didn't meet criteria		
62	30541093	2018	Clinical study	Other studies didn't meet criteria		
63	22649263	2012	Clinical study	Other studies didn't meet criteria		
64	38526814	2023	Clinical study	Other studies didn't meet criteria		
65	27966574	2016	Clinical study	Other studies didn't meet criteria		
66	34399704	2021	Clinical study	Other studies didn't meet criteria		
67	32159510	2020	Clinical study	Other studies didn't meet criteria		
68	28931089	2017	Clinical study	Other studies didn't meet criteria		
69	27821203	2017	Clinical study	Other studies didn't meet criteria		
70	26100928	2016	Clinical study	Other studies didn't meet criteria		
71	30901505	2019	Clinical study	Other studies didn't meet criteria		
72	29973712	2018	Clinical study	Other studies didn't meet criteria		
73	32036893	2020	Clinical study	Other studies didn't meet criteria		
74	36297053	2022	Clinical study	Other studies didn't meet criteria		
75	32744583	2020	Clinical study	Other studies didn't meet criteria		
76	30358831	2018	Clinical study	Other studies didn't meet criteria		
77	30735238	2019	Clinical study	Other studies didn't meet criteria		
78	31965846	2019	Clinical study	Other studies didn't meet criteria		
79	33331911	2021	Clinical study	Other studies didn't meet criteria		
80	32939005	2020	Clinical study	Other studies didn't meet criteria		
81	30732197	2019	Clinical study	Other studies didn't meet criteria		
82	31972239	2020	Clinical study	Other studies didn't meet criteria		
83	28553274	2017	Clinical study	Other studies didn't meet criteria		
84	35536006	2022	Clinical study	Other studies didn't meet criteria		
85	32936862	2020	Clinical study	Other studies didn't meet criteria		
86	28125762	2017	Clinical study	Other studies didn't meet criteria		
87	35357559	2022	Clinical study	Other studies didn't meet criteria		
88	37819145	2023	Clinical study	Other studies didn't meet criteria		
89	27504897	2016	Clinical study	Other studies didn't meet criteria		
90	34437440	2021	Clinical study	Other studies didn't meet criteria		
91	30794085	2020	Clinical study	Other studies didn't meet criteria		
92	32981834	2021	Clinical study	Other studies didn't meet criteria		
93	38517129	2024	Clinical study	Other studies didn't meet criteria		
94	30212253	2018	Clinical study	Other studies didn't meet criteria		
95	35057534	2022	Clinical study	Other studies didn't meet criteria		
96	30404694	2018	Clinical study	Other studies didn't meet criteria		
97	33593778	2021	Clinical study	Other studies didn't meet criteria		
98	32595645	2020	Clinical study	Other studies didn't meet criteria		
99	25435420	2015	Clinical study	Other studies didn't meet criteria		
100	31172651	2019	Clinical study	Other studies didn't meet criteria		
101	32666807	2021	Clinical study	Other studies didn't meet criteria		
102	27744545	2018	Clinical study	Other studies didn't meet criteria		
103	29198188	2017	Clinical study	Other studies didn't meet criteria		
104	33722860	2021	Clinical study	Other studies didn't meet criteria		
105	34835999	2021	Clinical study	Other studies didn't meet criteria		
106	36730730	2023	Clinical study	Other studies didn't meet criteria		
107	28157671	2016	Clinical study	Other studies didn't meet criteria		
108	27502158	2016	Clinical study	Other studies didn't meet criteria		
109	32871870	2020	Clinical study	Other studies didn't meet criteria		
110	38159728	2024	Clinical study	Other studies didn't meet criteria		
111	31798864	2019	Clinical study	Other studies didn't meet criteria		

112	35984175	2022	Clinical study	Other studies didn't meet criteria		
113	34444999	2021	Clinical study	Other studies didn't meet criteria		
114	35144523	2022	Clinical study	Other studies didn't meet criteria		
115	29506183	2018	Clinical study	Other studies didn't meet criteria		
116	33959381	2021	Clinical study	Other studies didn't meet criteria		
117	26919743	2016	Clinical study	Other studies didn't meet criteria		
118	31132957	2021	Clinical study	Other studies didn't meet criteria		
119	30399404	2019	Clinical study	Other studies didn't meet criteria		
120	31556132	2019	Clinical study	Other studies didn't meet criteria		
121	33103260	2020	Clinical study	Other studies didn't meet criteria		
122	31253623	2019	Clinical study	Other studies didn't meet criteria		
123	28418214	2017	Clinical study	Other studies didn't meet criteria		
124	22241860	2012	Clinical study	Other studies didn't meet criteria		
125	29274690	2018	Clinical study	Other studies didn't meet criteria		
126	31053143	2019	Clinical study	Other studies didn't meet criteria		
127	35372105	2022	Clinical study	Other studies didn't meet criteria		
128	34836348	2021	Clinical study	Other studies didn't meet criteria		
129	32528425	2020	Clinical study	Other studies didn't meet criteria		
130	33333806	2020	Clinical study	Other studies didn't meet criteria		
131	26549775	2016	Clinical study	Other studies didn't meet criteria		
132	32401138	2020	Clinical study	Other studies didn't meet criteria		
133	33580637	2021	Clinical study	Other studies didn't meet criteria		
134	28940737	2017	Clinical study	Other studies didn't meet criteria		
135	27018166	2016	Clinical study	Other studies didn't meet criteria		
136	31240835	2019	Clinical study	Other studies didn't meet criteria		
137	35458108	2022	Clinical study	Other studies didn't meet criteria		
138	31194939	2019	Clinical study	Other studies didn't meet criteria		
139	30471188	2019	Clinical study	Other studies didn't meet criteria		
140	31370734	2019	Clinical study	Other studies didn't meet criteria		
141	29350768	2018	Clinical study	Other studies didn't meet criteria		
142	36837890	2023	Clinical study	Other studies didn't meet criteria		
143	34776340	2022	Clinical study	Other studies didn't meet criteria		
144	36828255	2023	Clinical study	Other studies didn't meet criteria		
145	35725139	2022	Clinical study	Other studies didn't meet criteria		
146	33871591	2021	Clinical study	Other studies didn't meet criteria		
147	36986142	2023	Clinical study	Other studies didn't meet criteria		
148	32854216	2020	Clinical study	Other studies didn't meet criteria		
149	29464347	2019	Clinical study	Other studies didn't meet criteria		
150	33872583	2021	Clinical study	Other studies didn't meet criteria		
151	38244236	2024	Clinical study	Other studies didn't meet criteria		
152	34199383	2021	Clinical study	Other studies didn't meet criteria		
153	32994521	2020	Clinical study	Other studies didn't meet criteria		
154	34140026	2021	Clinical study	Other studies didn't meet criteria		
155	32475373	2020	Clinical study	Other studies didn't meet criteria		
156	35277019	2022	Clinical study	Other studies didn't meet criteria		
157	32414035	2020	Clinical study	Other studies didn't meet criteria		
158	31291462	2019	Clinical study	Other studies didn't meet criteria		
159	33092271	2020	Clinical study	Other studies didn't meet criteria		
160	36904207	2023	Clinical study	Other studies didn't meet criteria		
161	36698463	2023	Clinical study	Other studies didn't meet criteria		
162	34527515	2021	Clinical study	Other studies didn't meet criteria		
163	35271591	2022	Clinical study	Other studies didn't meet criteria		
164	28555008	2017	Clinical study	Other studies didn't meet criteria		
165	31615057	2019	Clinical study	Other studies didn't meet criteria		
166	37711620	2023	Clinical study	Other studies didn't meet criteria		
167	34531508	2021	Clinical study	Other studies didn't meet criteria		
168	34458156	2021	Clinical study	Other studies didn't meet criteria		
169	34836120	2021	Clinical study	Other studies didn't meet criteria		
170	38542697	2024	Clinical study	Other studies didn't meet criteria		
171	30696735	2019	Clinical study	Other studies didn't meet criteria		
172	34113697	2021	Clinical study	Other studies didn't meet criteria		
173	30803510	2019	Clinical study	Other studies didn't meet criteria		
174	34199006	2021	Clinical study	Other studies didn't meet criteria		
175	26562532	2015	Clinical study	Other studies didn't meet criteria		
176	32992898	2020	Clinical study	Other studies didn't meet criteria		
177	27782071	2016	Clinical study	Other studies didn't meet criteria		
178	32120990	2020	Clinical study	Other studies didn't meet criteria		
179	32722424	2020	Clinical study	Other studies didn't meet criteria		
180	33461965	2021	Clinical study	Other studies didn't meet criteria		
181	32708991	2020	Clinical study	Other studies didn't meet criteria		
182	37743489	2023	Clinical study	Other studies didn't meet criteria		
183	33505154	2021	Clinical study	Other studies didn't meet criteria		
184	30664020	2019	Clinical study	Other studies didn't meet criteria		
185	34444852	2021	Clinical study	Other studies didn't meet criteria		
186	31902372	2020	Clinical study	Other studies didn't meet criteria		
187	35745216	2022	Clinical study	Other studies didn't meet criteria		
188	34020445	2021	Clinical study	Other studies didn't meet criteria		
189	38419068	2024	Clinical study	Other studies didn't meet criteria		
190	34204572	2021	Clinical study	Other studies didn't meet criteria		
191	37272479	2023	Clinical study	Other studies didn't meet criteria		
192	35417758	2022	Clinical study	Other studies didn't meet criteria		
193	33561964	2021	Clinical study	Other studies didn't meet criteria		
194	35832045	2022	Clinical study	Other studies didn't meet criteria		
195	35322958	2022	Clinical study	Other studies didn't meet criteria		
196	31517324	2019	Clinical study	Other studies didn't meet criteria		
197	32860791	2021	Clinical study	Other studies didn't meet criteria		

198	29453751	2019	Clinical study	Other studies didn't meet criteria		
199	35182101	2022	Clinical study	Other studies didn't meet criteria		
200	32283870	2020	Clinical study	Other studies didn't meet criteria		
201	28230784	2017	Clinical study	Other studies didn't meet criteria		
202	26940357	2017	Clinical study	Other studies didn't meet criteria		
203	37705730	2023	Clinical study	Other studies didn't meet criteria		
204	30502656	2019	Clinical study	Other studies didn't meet criteria		
205	32235930	2020	Clinical study	Other studies didn't meet criteria		
206	37898434	2023	Clinical study	Other studies didn't meet criteria		
207	28069076	2016	Clinical study	Other studies didn't meet criteria		
208	34869782	2021	Clinical study	Other studies didn't meet criteria		
209	33557667	2021	Clinical study	Other studies didn't meet criteria		
210	35562794	2022	Clinical study	Other studies didn't meet criteria		
211	32798072	2020	Clinical study	Other studies didn't meet criteria		
212	31971861	2020	Clinical study	Other studies didn't meet criteria		
213	35651056	2022	Clinical study	Other studies didn't meet criteria		
214	36780542	2023	Clinical study	Other studies didn't meet criteria		
215	32703186	2020	Clinical study	Other studies didn't meet criteria		
216	33648648	2021	Clinical study	Other studies didn't meet criteria		
217	28639601	2017	Clinical study	Other studies didn't meet criteria		
218	31451722	2019	Clinical study	Other studies didn't meet criteria		
219	24690120	2014	Clinical study	Other studies didn't meet criteria		
220	31710785	2019	Clinical study	Other studies didn't meet criteria		
221	33021315	2021	Clinical study	Other studies didn't meet criteria		
222	28814395	2017	Clinical study	Other studies didn't meet criteria		
223	37699401	2023	Clinical study	Other studies didn't meet criteria		
224	36774101	2022	Clinical study	Other studies didn't meet criteria		
225	32028108	2020	Clinical study	Other studies didn't meet criteria		
226	31187868	2019	Clinical study	Other studies didn't meet criteria		
227	31815647	2019	Clinical study	Other studies didn't meet criteria		
228	34713713	2021	Clinical study	Other studies didn't meet criteria		
229	31377126	2019	Clinical study	Other studies didn't meet criteria		
230	36289300	2023	Clinical study	Other studies didn't meet criteria		
231	34851178	2021	Clinical study	Other studies didn't meet criteria		
232	34960094	2021	Clinical study	Other studies didn't meet criteria		
233	31991794	2020	Clinical study	Other studies didn't meet criteria		
234	34923911	2022	Clinical study	Other studies didn't meet criteria		
235	30400238	2018	Clinical study	Other studies didn't meet criteria		
236	25332326	2014	Clinical study	Other studies didn't meet criteria		
237	26426642	2015	Clinical study	Other studies didn't meet criteria		
238	33642191	2021	Clinical study	Other studies didn't meet criteria		
239	33238618	2020	Clinical study	Other studies didn't meet criteria		
240	30144429	2018	Clinical study	Other studies didn't meet criteria		
241	33933727	2021	Clinical study	Other studies didn't meet criteria		
242	36751856	2023	Clinical study	Other studies didn't meet criteria		
243	28902124	2017	Clinical study	Other studies didn't meet criteria		
244	31434096	2020	Clinical study	Other studies didn't meet criteria		
245	28591831	2017	Clinical study	Other studies didn't meet criteria		
246	32674247	2020	Clinical study	Other studies didn't meet criteria		
247	23038174	2013	Clinical study	Other studies didn't meet criteria		
248	36471554	2023	Clinical study	Other studies didn't meet criteria		
249	28677210	2017	Clinical study	Other studies didn't meet criteria		
250	33769467	2021	Clinical study	Other studies didn't meet criteria		
251	26951067	2016	Clinical study	Other studies didn't meet criteria		
252	35093031	2022	Clinical study	Other studies didn't meet criteria		
253	28806487	2017	Clinical study	Other studies didn't meet criteria		
254	34849762	2021	Clinical study	Other studies didn't meet criteria		
255	33387350	2021	Clinical study	Other studies didn't meet criteria		
256	32784721	2020	Clinical study	Other studies didn't meet criteria		
257	29482339	2018	Clinical study	Other studies didn't meet criteria		
258	32062351	2020	Clinical study	Other studies didn't meet criteria		
259	29989465	2019	Clinical study	Other studies didn't meet criteria		
260	32150694	2020	Clinical study	Other studies didn't meet criteria		
261	28285654	2017	Clinical study	Other studies didn't meet criteria		
262	35745166	2022	Clinical study	Other studies didn't meet criteria		
263	28531784	2016	Clinical study	Other studies didn't meet criteria		
264	34107331	2021	Clinical study	Other studies didn't meet criteria		
265	31996717	2020	Clinical study	Other studies didn't meet criteria		
266	30948911	2019	Clinical study	Other studies didn't meet criteria		
267	35906334	2023	Clinical study	Other studies didn't meet criteria		
268	32231240	2020	Clinical study	Other studies didn't meet criteria		
269	34256014	2021	Clinical study	Other studies didn't meet criteria		
270	29307330	2018	Clinical study	Other studies didn't meet criteria		
271	31851320	2020	Clinical study	Other studies didn't meet criteria		
272	35029612	2022	Clinical study	Other studies didn't meet criteria		
273	30876826	2020	Clinical study	Other studies didn't meet criteria		
274	38527529	2024	Clinical study	Other studies didn't meet criteria		
275	36872018	2023	Clinical study	Other studies didn't meet criteria		
276	38035748	2023	Clinical study	Other studies didn't meet criteria		
277	30939160	2019	Clinical study	Other studies didn't meet criteria		
278	30991877	2019	Clinical study	Other studies didn't meet criteria		
279	29571566	2019	Clinical study	Other studies didn't meet criteria		
280	33568158	2021	Clinical study	Other studies didn't meet criteria		
281	29452584	2018	Clinical study	Other studies didn't meet criteria		
282	33682457	2021	Clinical study	Other studies didn't meet criteria		
283	26272781	2015	Clinical study	Other studies didn't meet criteria		

284	26416813	2016	Clinical study	Other studies didn't meet criteria		
285	27974055	2016	Clinical study	Other studies didn't meet criteria		
286	31012086	2019	Clinical study	Other studies didn't meet criteria		
287	34822375	2021	Clinical study	Other studies didn't meet criteria		
288	30836671	2019	Clinical study	Other studies didn't meet criteria		
289	32536023	2020	Clinical study	Other studies didn't meet criteria		
290	32528151	2020	Clinical study	Other studies didn't meet criteria		
291	32910902	2020	Clinical study	Other studies didn't meet criteria		
292	32302292	2020	Clinical study	Other studies didn't meet criteria		
293	27725146	2017	Clinical study	Other studies didn't meet criteria		
294	33087380	2020	Clinical study	Other studies didn't meet criteria		
295	29755475	2018	Clinical study	Other studies didn't meet criteria		
296	37709337	2023	Clinical study	Other studies didn't meet criteria		
297	31271261	2019	Clinical study	Other studies didn't meet criteria		
298	36908030	2023	Clinical study	Other studies didn't meet criteria		
299	26249791	2016	Clinical study	Other studies didn't meet criteria		
300	30669671	2019	Clinical study	Other studies didn't meet criteria		
301	30538180	2018	Clinical study	Other studies didn't meet criteria		
302	31722138	2019	Clinical study	Other studies didn't meet criteria		
303	25453395	2015	Clinical study	Other studies didn't meet criteria		
304	30264073	2018	Clinical study	Other studies didn't meet criteria		
305	32058642	2020	Clinical study	Other studies didn't meet criteria		
306	37630728	2023	Clinical study	Other studies didn't meet criteria		
307	32806561	2020	Clinical study	Other studies didn't meet criteria		
308	29098426	2018	Clinical study	Other studies didn't meet criteria		
309	31451009	2019	Clinical study	Other studies didn't meet criteria		
310	30527258	2018	Clinical study	Other studies didn't meet criteria		
311	32544852	2020	Clinical study	Other studies didn't meet criteria		
312	32019174	2020	Clinical study	Other studies didn't meet criteria		
313	36226673	2022	Clinical study	Other studies didn't meet criteria		
314	32453977	2020	Clinical study	Other studies didn't meet criteria		
315	35807848	2022	Clinical study	Other studies didn't meet criteria		
316	34444798	2021	Clinical study	Other studies didn't meet criteria		
317	34048241	2021	Clinical study	Other studies didn't meet criteria		
318	30003647	2018	Clinical study	Other studies didn't meet criteria		
319	28624933	2018	Clinical study	Other studies didn't meet criteria		
320	29653862	2019	Clinical study	Other studies didn't meet criteria		
321	25299601	2014	Clinical study	Other studies didn't meet criteria		
322	33022987	2020	Clinical study	Other studies didn't meet criteria		
323	36382178	2022	Clinical study	Other studies didn't meet criteria		
324	32041265	2020	Clinical study	Other studies didn't meet criteria		
325	33655334	2021	Clinical study	Other studies didn't meet criteria		
326	27572508	2016	Clinical study	Other studies didn't meet criteria		
327	37156688	2023	Clinical study	Other studies didn't meet criteria		
328	32947608	2020	Clinical study	Other studies didn't meet criteria		
329	35057440	2022	Clinical study	Other studies didn't meet criteria		
330	29600282	2018	Clinical study	Other studies didn't meet criteria		
331	32105814	2020	Clinical study	Other studies didn't meet criteria		
332	31854308	2019	Clinical study	Other studies didn't meet criteria		
333	31666478	2019	Clinical study	Other studies didn't meet criteria		
334	33317254	2021	Clinical study	Other studies didn't meet criteria		
335	36100953	2022	Clinical study	Other studies didn't meet criteria		
336	34114016	2021	Clinical study	Other studies didn't meet criteria		
337	32615913	2020	Clinical study	Other studies didn't meet criteria		
338	32081286	2020	Clinical study	Other studies didn't meet criteria		
339	32369976	2020	Clinical study	Other studies didn't meet criteria		
340	33937616	2021	Clinical study	Other studies didn't meet criteria		
341	33471054	2021	Clinical study	Other studies didn't meet criteria		
342	32186326	2020	Clinical study	Other studies didn't meet criteria		
343	33811041	2021	Clinical study	Other studies didn't meet criteria		
344	32977254	2020	Clinical study	Other studies didn't meet criteria		
345	31696774	2020	Clinical study	Other studies didn't meet criteria		
346	26859894	2016	Clinical study	Other studies didn't meet criteria		
347	37537786	2023	Clinical study	Other studies didn't meet criteria		
348	32825823	2020	Clinical study	Other studies didn't meet criteria		
349	25903259	2016	Clinical study	Other studies didn't meet criteria		
350	34098340	2021	Clinical study	Other studies didn't meet criteria		
351	34100082	2021	Clinical study	Other studies didn't meet criteria		
352	30938383	2019	Clinical study	Other studies didn't meet criteria		
353	32075887	2020	Clinical study	Other studies didn't meet criteria		
354	32708278	2020	Clinical study	Other studies didn't meet criteria		
355	30056386	2018	Clinical study	Other studies didn't meet criteria		
356	28937980	2017	Clinical study	Other studies didn't meet criteria		
357	27381339	2016	Clinical study	Other studies didn't meet criteria		
358	33504116	2021	Clinical study	Other studies didn't meet criteria		
359	34980006	2022	Clinical study	Other studies didn't meet criteria		
360	33432175	2021	Clinical study	Other studies didn't meet criteria		
361	37630778	2023	Clinical study	Other studies didn't meet criteria		
362	31841548	2019	Clinical study	Other studies didn't meet criteria		
363	35902900	2022	Clinical study	Other studies didn't meet criteria		
364	32199523	2020	Clinical study	Other studies didn't meet criteria		
365	36424459	2022	Clinical study	Other studies didn't meet criteria		
366	32899492	2020	Clinical study	Other studies didn't meet criteria		
367	27151248	2016	Clinical study	Other studies didn't meet criteria		
368	37183583	2024	Clinical study	Other studies didn't meet criteria		
369	32266159	2020	Clinical study	Other studies didn't meet criteria		

370	34092633	2021	Clinical study	Other studies didn't meet criteria		
371	35782954	2022	Clinical study	Other studies didn't meet criteria		
372	34245762	2021	Clinical study	Other studies didn't meet criteria		
373	32782162	2021	Clinical study	Other studies didn't meet criteria		
374	34922581	2021	Clinical study	Other studies didn't meet criteria		
375	35912804	2022	Clinical study	Other studies didn't meet criteria		
376	26511097	2016	Clinical study	Other studies didn't meet criteria		
377	26455903	2015	Clinical study	Other studies didn't meet criteria		
378	32899326	2020	Clinical study	Other studies didn't meet criteria		
379	36776602	2023	Clinical study	Other studies didn't meet criteria		
380	34910161	2022	Clinical study	Other studies didn't meet criteria		
381	30423561	2019	Clinical study	Other studies didn't meet criteria		
382	28988196	2018	Clinical study	Other studies didn't meet criteria		
383	27719686	2016	Clinical study	Other studies didn't meet criteria		
384	32626450	2019	Clinical study	Other studies didn't meet criteria		
385	32742642	2020	Clinical study	Other studies didn't meet criteria		
386	33313854	2021	Clinical study	Other studies didn't meet criteria		
387	34114015	2021	Clinical study	Other studies didn't meet criteria		
388	34836127	2021	Clinical study	Other studies didn't meet criteria		
389	34444866	2021	Clinical study	Other studies didn't meet criteria		
390	32956679	2021	Clinical study	Other studies didn't meet criteria		
391	27288567	2016	Clinical study	Other studies didn't meet criteria		
392	34617562	2022	Clinical study	Other studies didn't meet criteria		
393	29490103	2018	Clinical study	Other studies didn't meet criteria		
394	31317029	2019	Clinical study	Other studies didn't meet criteria		
395	30139999	2018	Clinical study	Other studies didn't meet criteria		
396	31994568	2020	Clinical study	Other studies didn't meet criteria		
397	32300799	2021	Clinical study	Other studies didn't meet criteria		
398	27810310	2016	Clinical study	Other studies didn't meet criteria		
399	36296911	2022	Clinical study	Other studies didn't meet criteria		
400	31851298	2020	Clinical study	Other studies didn't meet criteria		
401	29719871	2018	Clinical study	Other studies didn't meet criteria		
402	31547446	2019	Clinical study	Other studies didn't meet criteria		
403	34579043	2021	Clinical study	Other studies didn't meet criteria		
404	36462977	2023	Clinical study	Other studies didn't meet criteria		
405	37630452	2023	Clinical study	Other studies didn't meet criteria		
406	36265810	2022	Clinical study	Other studies didn't meet criteria		
407	32438623	2020	Clinical study	Other studies didn't meet criteria		
408	26599039	2016	Clinical study	Other studies didn't meet criteria		
409	34560884	2021	Clinical study	Other studies didn't meet criteria		
410	33177712	2020	Clinical study	Other studies didn't meet criteria		
411	28164731	2017	Clinical study	Other studies didn't meet criteria		
412	29332901	2017	Clinical study	Other studies didn't meet criteria		
413	26460205	2016	Clinical study	Other studies didn't meet criteria		
414	31811045	2020	Clinical study	Other studies didn't meet criteria		
415	38151533	2024	Clinical study	Other studies didn't meet criteria		
416	38542685	2024	Clinical study	Other studies didn't meet criteria		
417	31520001	2019	Clinical study	Other studies didn't meet criteria		
418	33842951	2021	Clinical study	Other studies didn't meet criteria		
419	28178201	2017	Clinical study	Other studies didn't meet criteria		
420	31551328	2019	Clinical study	Other studies didn't meet criteria		
421	31829172	2019	Clinical study	Other studies didn't meet criteria		
422	31371751	2019	Clinical study	Other studies didn't meet criteria		
423	36604516	2023	Clinical study	Other studies didn't meet criteria		
424	30636111	2019	Clinical study	Other studies didn't meet criteria		
425	29867803	2018	Clinical study	Other studies didn't meet criteria		
426	26265295	2015	Clinical study	Other studies didn't meet criteria		
427	38426663	2024	Clinical study	Other studies didn't meet criteria		
428	29378051	2018	Clinical study	Other studies didn't meet criteria		
429	34078289	2021	Clinical study	Other studies didn't meet criteria		
430	27606833	2017	Clinical study	Other studies didn't meet criteria		
431	29936607	2018	Clinical study	Other studies didn't meet criteria		
432	31043597	2019	Clinical study	Other studies didn't meet criteria		
433	29624599	2018	Clinical study	Other studies didn't meet criteria		
434	32620337	2020	Clinical study	Other studies didn't meet criteria		
435	34759297	2021	Clinical study	Other studies didn't meet criteria		
436	30867328	2019	Clinical study	Other studies didn't meet criteria		
437	34010651	2021	Clinical study	Other studies didn't meet criteria		
438	28821315	2017	Clinical study	Other studies didn't meet criteria		
439	32073296	2020	Clinical study	Other studies didn't meet criteria		
440	22699609	2012	Clinical study	Other studies didn't meet criteria		
441	32925728	2020	Clinical study	Other studies didn't meet criteria		
442	36651990	2023	Clinical study	Other studies didn't meet criteria		
443	20668239	2011	Clinical study	Other studies didn't meet criteria		
444	27510655	2017	Clinical study	Other studies didn't meet criteria		
445	25258407	2014	Clinical study	Other studies didn't meet criteria		
446	32033224	2020	Clinical study	Other studies didn't meet criteria		
447	34795283	2021	Clinical study	Other studies didn't meet criteria		
448	28971851	2017	Clinical study	Other studies didn't meet criteria		
449	34958387	2022	Clinical study	Other studies didn't meet criteria		
450	36768394	2023	Clinical study	Other studies didn't meet criteria		
451	31963239	2020	Clinical study	Other studies didn't meet criteria		
452	30888537	2019	Clinical study	Other studies didn't meet criteria		
453	30698687	2019	Clinical study	Other studies didn't meet criteria		
454	27708392	2016	Clinical study	Other studies didn't meet criteria		
455	31235863	2019	Clinical study	Other studies didn't meet criteria		

456	27500753	2016	Clinical study	Other studies didn't meet criteria		
457	29873593	2018	Clinical study	Other studies didn't meet criteria		
458	32779963	2020	Clinical study	Other studies didn't meet criteria		
459	28228424	2017	Clinical study	Other studies didn't meet criteria		
460	31136662	2019	Clinical study	Other studies didn't meet criteria		
461	35231853	2022	Clinical study	Other studies didn't meet criteria		
462	33374578	2020	Review 1	Not-original research		
463	28390574	2017	Review 2	Not-original research		
464	36907413	2023	Review 3	Not-original research		
465	35208201	2022	Review 4	Not-original research		
466	33630874	2021	Review 5	Not-original research		
467	27272325	2016	Review 6	Not-original research		
468	29781841	2018	Review 7	Not-original research		
469	34175022	2021	Review 8	Not-original research		
470	38116074	2023	Review 9	Not-original research		
471	32289261	2020	Review 10	Not-original research		
472	24995004	2014	Review 11	Not-original research		
473	30661321	2019	Review 12	Not-original research		
474	34959933	2021	Review 13	Not-original research		
475	31964203	2020	Review 14	Not-original research		
476	32211860	2020	Review 15	Not-original research		
477	33590719	2021	Review 16	Not-original research		
478	27712080	2018	Review 17	Not-original research		
479	29757343	2018	Review 18	Not-original research		
480	34201611	2021	Review 19	Not-original research		
481	32443535	2020	Review 20	Not-original research		
482	37447285	2023	Review 21	Not-original research		
483	29925774	2018	Review 22	Not-original research		
484	26762459	2016	Review 23	Not-original research		
485	33945146	2021	Review 24	Not-original research		
486	27088328	2016	Review 25	Not-original research		
487	30984765	2019	Review 26	Not-original research		
488	34203776	2021	Review 27	Not-original research		
489	33114017	2020	Review 28	Not-original research		
490	27824805	2016	Review 29	Not-original research		
491	29489753	2018	Review 30	Not-original research		
492	31373365	2019	Review 31	Not-original research		
493	30773108	2019	Review 32	Not-original research		
494	34311029	2021	Review 33	Not-original research		
495	37949114	2024	Review 34	Not-original research		
496	37298483	2023	Review 35	Not-original research		
497	29493330	2018	Review 36	Not-original research		
498	36013376	2022	Review 37	Not-original research		
499	26578751	2015	Review 38	Not-original research		
500	38516317	2024	Review 39	Not-original research		
501	31473156	2020	Review 40	Not-original research		
502	31811292	2020	Review 41	Not-original research		
503	25758642	2015	Review 42	Not-original research		
504	30612189	2019	Review 43	Not-original research		
505	37968837	2023	Review 44	Not-original research		
506	32986319	2020	Review 45	Not-original research		
507	33422403	2021	Review 46	Not-original research		
508	32979504	2020	Review 47	Not-original research		
509	29032502	2018	Review 48	Not-original research		
510	30479462	2018	Review 49	Not-original research		
511	31474424	2019	Review 50	Not-original research		
512	37774217	2023	Review 51	Not-original research		
513	34444674	2021	Review 52	Not-original research		
514	37085614	2023	Review 53	Not-original research		
515	33724115	2022	Review 54	Not-original research		
516	33149306	2020	Review 55	Not-original research		
517	37513682	2023	Review 56	Not-original research		
518	36026526	2022	Review 57	Not-original research		
519	25484891	2014	Review 58	Not-original research		
520	35697155	2022	Review 59	Not-original research		
521	38174642	2024	Review 60	Not-original research		
522	32867808	2020	Review 61	Not-original research		
523	32388710	2020	Review 62	Not-original research		
524	32024556	2020	Review 63	Not-original research		
525	31140389	2019	Review 64	Not-original research		
526	38448632	2024	Review 65	Not-original research		
527	36611827	2022	Review 66	Not-original research		
528	30489399	2019	Review 67	Not-original research		
529	36102345	2023	Review 68	Not-original research		
530	31631671	2020	Review 69	Not-original research		
531	32818934	2021	Review 70	Not-original research		
532	34209683	2021	Review 71	Not-original research		
533	30936547	2019	Review 72	Not-original research		
534	31412673	2019	Review 73	Not-original research		
535	32985923	2020	Review 74	Not-original research		
536	32267865	2020	Review 75	Not-original research		
537	35010920	2021	Review 76	Not-original research		
538	35947353	2023	Review 77	Not-original research		
539	32002758	2020	Review 78	Not-original research		
540	28986601	2018	Review 79	Not-original research		
541	32549225	2020	Review 80	Not-original research		

542	25851728	2015	Review 81	Not-original research		
543	35304983	2022	Review 82	Not-original research		
544	28936910	2017	Review 83	Not-original research		
545	30394894	2019	Review 84	Not-original research		
546	37050953	2023	Review 85	Not-original research		
547	29852087	2018	Review 86	Not-original research		
548	29766369	2019	Review 87	Not-original research		
549	32781516	2020	Review 88	Not-original research		
550	26607554	2015	Review 89	Not-original research		
551	33613480	2021	Review 90	Not-original research		
552	32610641	2020	Review 91	Not-original research		
553	26514720	2015	Review 92	Not-original research		
554	23594389	2013	Review 93	Not-original research		
555	29109959	2017	Review 94	Not-original research		
556	30747307	2019	Review 95	Not-original research		
557	37172326	2023	Review 96	Not-original research		
558	32174888	2020	Review 97	Not-original research		
559	35219904	2022	Review 98	Not-original research		
560	25584460	2015	Review 99	Not-original research		
561	26269668	2015	Review 100	Not-original research		
562	34836399	2021	Review 101	Not-original research		
563	33271210	2021	Review 102	Not-original research		
564	35708057	2023	Review 103	Not-original research		
565	24416266	2014	Review 104	Not-original research		
566	32988391	2020	Review 105	Not-original research		
567	38638485	2024	Review 106	Not-original research		
568	30052094	2018	Review 107	Not-original research		
569	29114246	2017	Review 108	Not-original research		
570	34371912	2021	Review 109	Not-original research		
571	28524627	2017	Review 110	Not-original research		
572	31255903	2019	Review 111	Not-original research		
573	33324864	2020	Fundamental research	Non-clinical studies		
574	34962977	2021	Fundamental research	Non-clinical studies		
575	33765135	2021	Fundamental research	Non-clinical studies		
576	29893876	2018	Fundamental research	Non-clinical studies		
577	33694368	2021	Fundamental research	Non-clinical studies		
578	36271770	2023	Fundamental research	Non-clinical studies		
579	30422704	2019	Fundamental research	Non-clinical studies		
580	33349590	2021	Fundamental research	Non-clinical studies		
581	31965839	2019	Fundamental research	Non-clinical studies		
582	37098952	2023	Fundamental research	Non-clinical studies		
583	32592577	2020	Fundamental research	Non-clinical studies		
584	30355801	2018	Fundamental research	Non-clinical studies		
585	34398307	2021	Fundamental research	Non-clinical studies		
586	19043404	2009	Fundamental research	Non-clinical studies		
587	27038949	2016	Fundamental research	Non-clinical studies		
588	31582724	2019	Fundamental research	Non-clinical studies		
589	28558792	2017	Fundamental research	Non-clinical studies		
590	33952353	2021	Fundamental research	Non-clinical studies		
591	36348807	2022	Fundamental research	Non-clinical studies		
592	36471455	2022	Fundamental research	Non-clinical studies		
593	34587256	2021	Fundamental research	Non-clinical studies		
594	31634399	2019	Fundamental research	Non-clinical studies		
595	34509665	2021	Fundamental research	Non-clinical studies		
596	33138291	2020	Fundamental research	Non-clinical studies		
597	29511074	2018	Fundamental research	Non-clinical studies		
598	33320182	2021	Fundamental research	Non-clinical studies		
599	32564858	2020	Fundamental research	Non-clinical studies		
600	37624284	2023	Fundamental research	Non-clinical studies		
601	33710512	2021	Fundamental research	Non-clinical studies		
602	35965387	2022	Fundamental research	Non-clinical studies		
603	36617268	2023	Fundamental research	Non-clinical studies		
604	37011713	2023	Fundamental research	Non-clinical studies		
605	33480132	2021	Fundamental research	Non-clinical studies		
606	23075436	2012	Fundamental research	Non-clinical studies		
607	38587063	2024	Fundamental research	Non-clinical studies		
608	31373737	2019	Fundamental research	Non-clinical studies		
609	37829353	2023	Fundamental research	Non-clinical studies		
610	34784923	2021	Fundamental research	Non-clinical studies		
611	33454743	2021	Fundamental research	Non-clinical studies		
612	37869782	2023	Fundamental research	Non-clinical studies		
613	32155205	2020	Fundamental research	Non-clinical studies		
614	37740322	2023	Fundamental research	Non-clinical studies		
615	32857814	2020	Fundamental research	Non-clinical studies		
616	32166413	2020	Fundamental research	Non-clinical studies		
617	32194221	2020	Fundamental research	Non-clinical studies		
618	27633737	2016	Fundamental research	Non-clinical studies		
619	35698917	2022	Fundamental research	Non-clinical studies		
620	32337713	2020	Fundamental research	Non-clinical studies		
621	33304337	2020	Fundamental research	Non-clinical studies		
622	36858136	2023	Fundamental research	Non-clinical studies		
623	28744326	2017	Fundamental research	Non-clinical studies		
624	31162138	2019	Fundamental research	Non-clinical studies		
625	30998992	2019	Fundamental research	Non-clinical studies		
626	27518814	2016	Fundamental research	Non-clinical studies		
627	29267377	2017	Fundamental research	Non-clinical studies		

628	38633690	2024	Fundamental research	Non-clinical studies		
629	34253098	2021	Fundamental research	Non-clinical studies		
630	26862979	2016	Fundamental research	Non-clinical studies		
631	34959997	2021	Fundamental research	Non-clinical studies		
632	27585552	2016	Fundamental research	Non-clinical studies		
633	29558912	2018	Fundamental research	Non-clinical studies		
634	37766696	2023	Fundamental research	Non-clinical studies		
635	31238287	2019	Fundamental research	Non-clinical studies		
636	31761826	2020	Fundamental research	Non-clinical studies		
637	32712897	2021	Fundamental research	Non-clinical studies		
638	33775867	2021	Fundamental research	Non-clinical studies		
639	35915514	2022	Fundamental research	Non-clinical studies		
640	34684436	2021	Fundamental research	Non-clinical studies		
641	23898195	2013	Fundamental research	Non-clinical studies		
642	34070816	2021	Fundamental research	Non-clinical studies		
643	28873671	2017	Fundamental research	Non-clinical studies		
644	25418803	2015	Fundamental research	Non-clinical studies		
645	30827566	2019	Fundamental research	Non-clinical studies		
646	36499541	2022	Fundamental research	Non-clinical studies		
647	33177795	2020	Fundamental research	Non-clinical studies		
648	25438151	2014	Fundamental research	Non-clinical studies		
649	37083181	2023	Fundamental research	Non-clinical studies		
650	20962874	2011	Fundamental research	Non-clinical studies		
651	20339542	2010	Fundamental research	Non-clinical studies		
652	34178715	2021	Fundamental research	Non-clinical studies		
653	30867067	2019	Fundamental research	Non-clinical studies		
654	32412900	2020	Fundamental research	Non-clinical studies		
655	38553986	2024	Fundamental research	Non-clinical studies		
656	34774538	2022	Fundamental research	Non-clinical studies		
657	32303546	2020	Fundamental research	Non-clinical studies		
658	28245817	2017	Fundamental research	Non-clinical studies		
659	30006229	2018	Fundamental research	Non-clinical studies		
660	30665298	2019	Fundamental research	Non-clinical studies		
661	36177023	2022	Fundamental research	Non-clinical studies		
662	25714718	2015	Fundamental research	Non-clinical studies		
663	37602405	2023	Fundamental research	Non-clinical studies		
664	32862023	2020	Fundamental research	Non-clinical studies		
665	33435396	2021	Fundamental research	Non-clinical studies		
666	35863030	2022	Fundamental research	Non-clinical studies		
667	32105727	2020	Fundamental research	Non-clinical studies		
668	34497333	2021	Fundamental research	Non-clinical studies		
669	30550821	2019	Fundamental research	Non-clinical studies		
670	28655159	2017	Fundamental research	Non-clinical studies		
671	32806674	2020	Fundamental research	Non-clinical studies		
672	30826445	2019	a case series	Not-original research		
